# Molecular View on the *i*RGD Peptide Binding Mechanism: Implications for Integrin Activity and Selectivity Profiles

**DOI:** 10.1021/acs.jcim.3c01071

**Published:** 2023-10-03

**Authors:** Vincenzo Maria D’Amore, Greta Donati, Elena Lenci, Beatrice Stefanie Ludwig, Susanne Kossatz, Monica Baiula, Andrea Trabocchi, Horst Kessler, Francesco Saverio Di Leva, Luciana Marinelli

**Affiliations:** † Department of Pharmacy, Università degli Studi di Napoli “Federico II”, Via D. Montesano 49, 80131 Naples, Italy; ‡ Department of Chemistry “Ugo Schiff″, University of Florence, Via della Lastruccia 13, I-50019 Sesto Fiorentino, Florence, Italy; § Department of Nuclear Medicine, University Hospital Klinikum Rechts der Isar and Central Institute for Translational Cancer Research (TranslaTUM), Technical University Munich, Munich 81675, Germany; ∥ Department of Chemistry, Institute for Advanced Study, Technical University Munich, Garching 85748, Germany; ⊥ Department of Pharmacy and Biotechnology, University of Bologna, Via Irnerio 48, 40126 Bologna, Italy

## Abstract

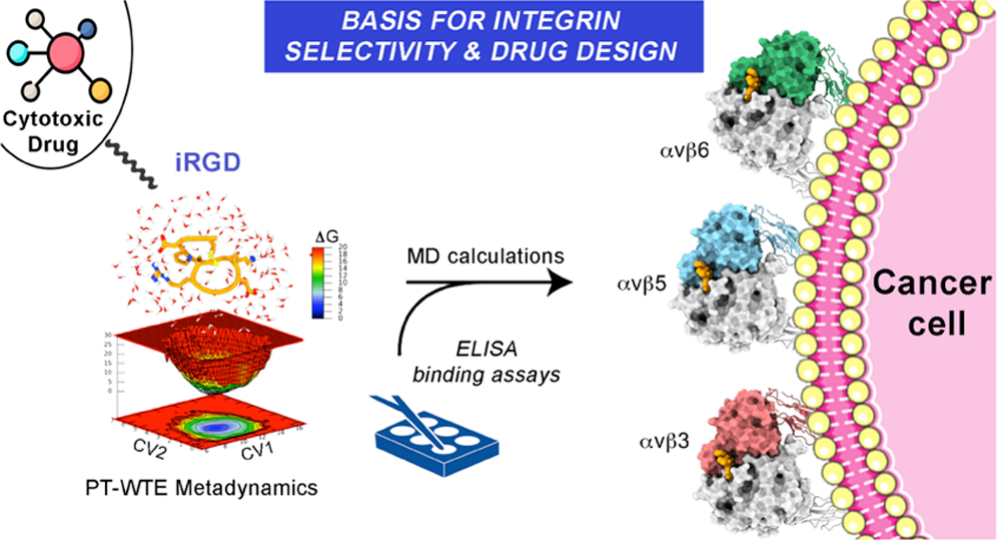

Receptor-selective peptides are widely used as smart carriers for specific tumor-targeted delivery. A remarkable example is the cyclic nonapeptide *i*RGD (CRGDKPGDC, **1**) that couples intrinsic cytotoxic effects with striking tumor-homing properties. These peculiar features are based on a rather complex multistep mechanism of action, where the primary event is the recognition of RGD integrins. Despite the high number of preclinical studies and the recent success of a phase I trial for the treatment of pancreatic ductal adenocarcinoma (PDAC), there is little information available about the *i*RGD three-dimensional (3D) structure and integrin binding properties. Here, we re-evaluate the peptide’s affinity for cancer-related integrins including not only the previously known targets αvβ3 and αvβ5 but also the αvβ6 isoform, which is known to drive cell growth, migration, and invasion in many malignancies including PDAC. Furthermore, we use parallel tempering in the well-tempered ensemble (PT-WTE) metadynamics simulations to characterize the in-solution conformation of *i*RGD and extensive molecular dynamics calculations to fully investigate its binding mechanism to integrin partners. Finally, we provide clues for fine-tuning the peptide’s potency and selectivity profile, which, in turn, may further improve its tumor-homing properties.

## 1 Introduction

**Chart 1:**
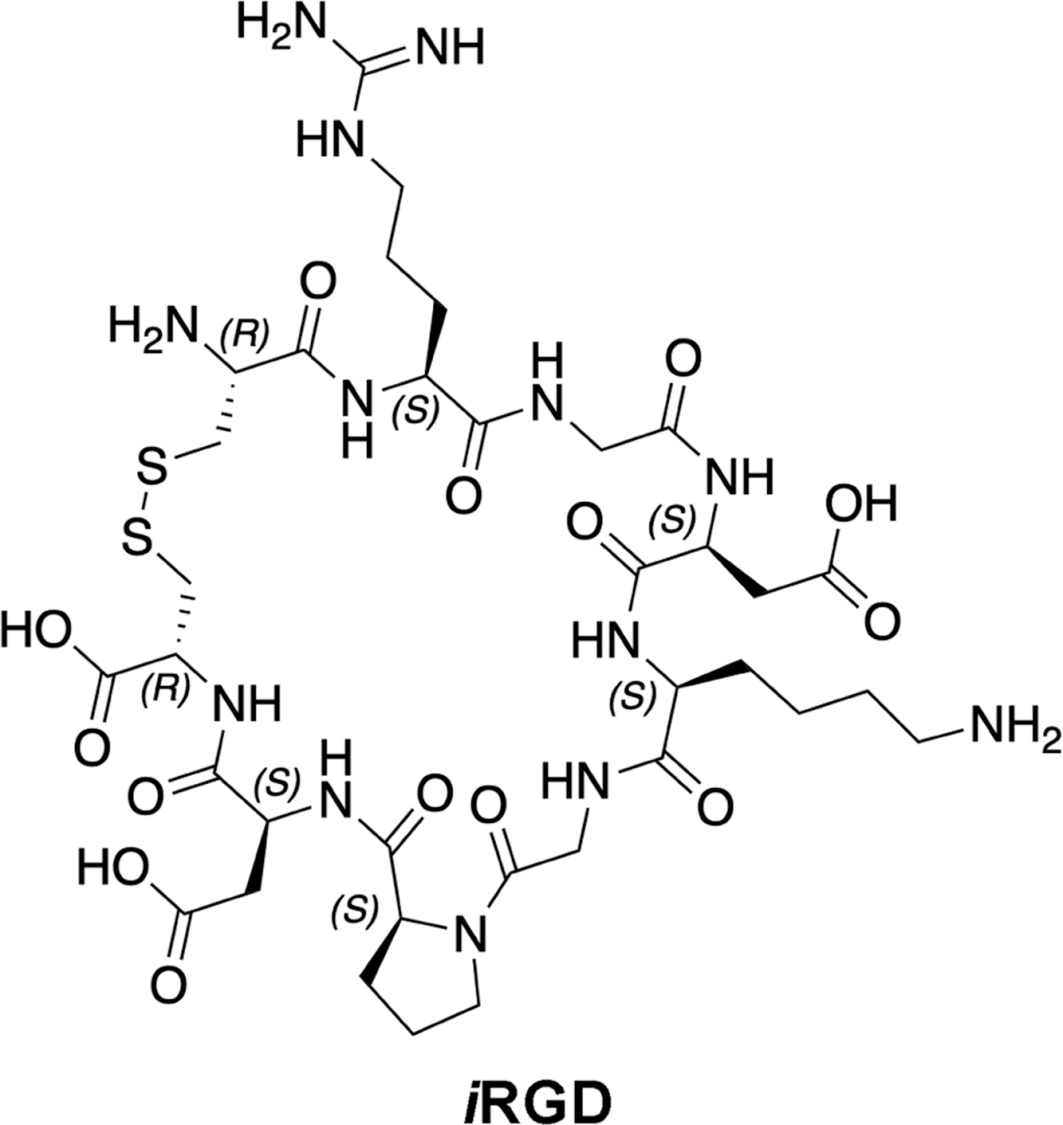
2D Chemical Structure of *i*RGD (**1**)

Nowadays, the clinical efficacy of chemotherapeutic drugs is frequently hampered either by a lack of selectivity over healthy cells or by poor pharmacokinetic properties, including cancer homing and penetration. This is especially true for solid tumors, which are frequently characterized by the upregulation of junction proteins such as desmoglein 2 (DSG2) and E-cadherin, and extracellular matrix (ECM) components (i.e., fibrinogen and collagen), which form a physical barrier against the intracellular transport of exogenous molecules.^1^ For these reasons, anticancer compounds often need to be administered at high doses to exert relevant pharmacological effects, with the rise of serious adverse reactions.^2−4^ A feasible solution to the tissue penetration problem is represented by smart carriers that can vehicle the desired drug to extravascular cancer tissue. Carriers of different natures have been developed such as gold nanoparticles,^5−7^ liposomes,^8,9^ polymer micelles,^10^ or receptor-selective peptides.^11−15^ In this context, Ruoslahti and co-workers identified an RGD integrins-targeting cyclic nonapeptide, namely, *i*RGD (internalizing RGD, CRGDKGPDC, **1**—Chart 1), endowed with remarkable tumor-homing properties.^16,17^ Notably, this peptide can improve the tumor penetration and efficacy of chemotherapeutics through two alternative mechanisms.^1,18−32^ In fact, *i*RGD can be either covalently bioconjugated—usually functionalizing the C-terminal Cys^9^—to organic and peptidic drugs or attached to the surface of other delivering systems like nanoparticles, liposomes, or oncolytic viruses.^18−32^ On the other hand, the tumor endocytosis of cytotoxic agents such as cisplatin, gemcitabine, doxorubicin, nab-paclitaxel, and trastuzumab is enhanced by the simple coadministration of **1**.^17,33,34^ As a result, hundreds of distinct applications involving *i*RGD have been published during the past decade,^18−35^ claiming the potential of this peptide as a game changer in the anticancer field.^36^ Worth of special note are the clinical results obtained by the combination of **1** with nab-paclitaxel and gemcitabine: a phase I trial reported a safe tolerability profile and a longer progression-free survival in the treatment of metastatic pancreatic ductal adenocarcinoma (PDAC), a neoplasia that is usually poorly susceptible to both traditional chemotherapy and immunotherapy.^34,37^

The striking *i*RGD’s tumor-homing activity is linked to the marked overexpression of RGD integrins on the cancer cell membrane.^38−40^ Notably, many peptides, peptidomimetics, and small molecules targeting these receptors have been used over the years, but none of them have shown tumor-penetrating properties comparable to **1**. This can be correlated to the peculiar multistage internalization process of *i*RGD, where the binding to integrins represents only the first step.^16^ In fact, once the peptide binds to the integrin receptor, it experiences a proteolytical cleavage at the Lys^5^–Gly^6^ bond that results in exposure and the following release of the cryptic C-terminal CRGDK sequence (CendR motif). The latter is a common recognition pattern of neuropilin-1 (NRP-1), a tyrosine kinases’ coreceptor playing multiple roles in angiogenesis, cell migration, and invasion.^41^ The binding of the CendR motif to NRP-1 triggers the internalization of the peptide–receptor complex and is responsible for the *i*RGD’s intrinsic cytotoxic effects.^42^ In this regard, in vitro experiments proved that *i*RGD can inhibit tumor migration and induce chemorepulsion based on a CendR- and NRP-1-dependent mechanism of action.^43^ The tropism and selectivity of **1** for cancer tissues are, however, ruled by its affinity for RGD integrins.^16^ So far, only the binding to the αvβ3 and αvβ5 isoforms has been demonstrated, whereas to our knowledge, no direct interaction data are currently available for other clinically relevant subtypes such as αvβ6. The latter recently came to the limelight for its involvement in the development of idiopathic pulmonary fibrosis (IPF) as well as several malignancies such as colorectal cancers and PDAC.^44−47^ Indeed, αvβ6 promotes in vitro PDAC cell growth, survival, migration, and invasion. Accordingly, the treatment of either αvβ6-positive human PDAC xenografts or transgenic mice with an αvβ6 blocking antibody combined with gemcitabine was shown to significantly reduce tumor growth while increasing the survival rate.^48^ Thus, we wondered if at least part of the peptide’s efficacy may be due to a still undetected affinity to αvβ6. To answer this question, we here extended the in vitro characterization of the *i*RGD’s integrin selectivity profile, repeating the IC_50_s measurements for the known cognate receptors αvβ3 and αvβ5 and demonstrating for the first time a mid-low nanomolar potency toward the αvβ6 isoform. Furthermore, the present article fills the knowledge gap on the structural basis of the *i*RGD–integrin interaction. Indeed, despite the potential clinical relevance of **1**,^34,37^ its solution conformation, interaction mode with integrin receptors, and structure–activity relationships are still unknown. In this perspective, we used a combination of bioinformatics and advanced biosimulations to provide a high-resolution description of the *i*RGD’s folding and mode of binding to the αvβ3, αvβ5, and αvβ6 integrins. The peptide’s affinity and selectivity profile were thus rationalized, also explaining how its proteolytic cleavage and bioconjugation (with cargos of different natures) can take place without altering the affinity for the target receptors. Our results help to understand in more detail the integrin binding properties of *i*RGD and provide valuable clues for fine-tuning its selectivity profile and, in turn, its tumor-homing properties.

## 2 Materials and Methods

### 2.1 Solid-Phase Integrin Binding Assay

*i*RGD and cilengitide^49,50^ were purchased as pure substances from MedChemExpress, while [RGD-Chg-E]-CONH_2_ (**2**) was synthesized according to a previously reported protocol.^51^ Purified αvβ3 (Sino Biological Europe GmbH) and αvβ5 (R&D Systems, Inc.) were diluted to 0.5 μg/mL, while αvβ6 (Sino Biological Europe GmbH) was diluted to 1 μg/mL in coating buffer containing Tris–HCl (20 mmol/L; pH 7.4), NaCl (150 mmol/L), MnCl_2_ (1 mmol/L), CaCl_2_ (2 mmol/L), and MgCl_2_ (1 mmol/L). An aliquot of diluted receptors (100 μL per well) was added to 96-well microtiter plates (Nunc MW96F Maxisorp Straight) and incubated overnight at 4 °C. The plates were then incubated with blocking solution (coating buffer plus 1% bovine serum albumin) for an additional 2 h at room temperature to block nonspecific binding, followed by 3 h incubation at room temperature with various concentrations of test compounds in the presence of, respectively, vitronectin (1 μg/mL, Sigma-Aldrich) for αvβ3 and αvβ5 and fibronectin for αvβ6 (1 μg/mL, Sigma-Aldrich) biotinylated using the EZ-Link Sulfo-NHS-Biotinylation kit (Thermo Fisher). After washing, the plates were incubated for 1 h at room temperature with the streptavidin–biotinylated peroxidase complex (GE Health), followed by 15 min incubation with substrate reagent solution (100 μL; R&D Systems) before stopping the reaction by addition of H_2_SO_4_ (0.25 M, 50 μL). Absorbance at 415 nm was read with a BMG Labtech Fluostar Optima microplate reader. All of the experiments were performed in triplicates, and the data collected were analyzed using the GraphPad 5.0 Software Package (GraphPad Prism, San Diego, CA).

### 2.2 PT-WTE Calculations

Parallel tempering in the well-tempered ensemble (WTE)^52^ is a powerful enhanced sampling method based on the combination of parallel tempering (PT) and well-tempered metadynamics (WT-MetaD).^53,54^ In PT, *n* copies of the system are simulated at different temperatures, with periodic coordinate exchanges attempted between adjacent replicas and ruled by the Metropolis–Hastings criterion. The general idea is that medium–high free energy barriers that trap low-temperature replicas in local energy minima can be crossed at higher temperatures. On the other hand, in MetaD, the sampling of the simulation is boosted by a history-dependent bias potential (*V*_G_), made of Gaussians deposited on a selected number of reaction coordinates (i.e., slow degrees of freedom) referred to as collective variables (CVs)
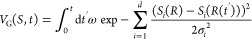
1where *S_i_* is the value of the *i*th CV, σ_*i*_ is the width of the Gaussian function, and ω is the rate at which the bias is deposited. In WT-MetaD, the bias deposition rate ω is exponentially rescaled over time depending on how much potential has already been added in the same region of the CV phase space, according to the following formula
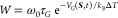
2where *W* is the Gaussian height, *k*_B_ is Boltzmann’s constant, ω_0_ is the initial energy rate, τ_G_ is the Gaussian deposition stride, and *V*_G_ (***S**, t*) is the bias potential accumulated in *S* over time *t*. Δ*T* is an input parameter with the dimension of a temperature, which controls how quickly the Gaussian height is decreased and is often written in terms of a so-called bias factor γ = (*T* + Δ*T*)/*T*. At the end of a WT-MetaD simulation, the deposited bias potential *V*_G_ asymptotically converges to the inverse value of a fraction of free energy *F*
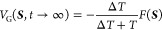
3When the potential energy is used as CV in WT-MetaD simulations, a well-defined distribution known as the well-tempered ensemble (WTE) is sampled. In WTE, the system experiences enhanced energy fluctuations that can be used to facilitate the exchange processes and reduce the number of replicas required for PT. In our case, the metadynamics Gaussians were deposited every 0.5 ps with a width of 145 kJ/mol and an initial height of 2.5 kJ/mol, which was gradually decreased based on a bias factor γ = 24. Then, 6 replicas were distributed according to the formula proposed by Prakash et al.^55^ to span the temperature interval 300–450 K. Each replica was simulated for 140 ns in the NVT ensemble using the stochastic rescaling thermostat.^56^ The coordinate exchanges were attempted every 0.5 ps, obtaining an average acceptance probability of 25% between all of the neighbor replicas. A further advantage of the WTE ensemble is that the canonical energy average is conserved, and all of the other canonical observables can be estimated a posteriori. Thus, the Tiwary–Parrinello reweighting scheme^57^ was employed to compute the free energy surface (FES) associated with the folding of **1** as a function of two selected CVs. The first one is the dihedral correlation (Dih_cor_) between all of the torsion angles of the peptide backbone, also including the peptide disulfide bridge
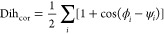
4where the ϕ*_i_* and ψ*_i_* values are the instantaneous values for the torsion angles of interest. This function measures the degree of similarity between adjacent dihedral angles and, if extended to the entire backbone, can describe global conformational changes. The second CV, H_bonds_, estimates the number of intramolecular backbone–backbone H-bonds
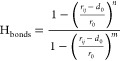
5where *i* and *j* are defined as all of the possible combinations between the amide hydrogen and oxygen atoms (except the C-terminal carboxyl oxygens) of the peptide backbone; *d*_0_ and *r*_0_ distances were set to 0 and 2.5 Å, while the *n* and *m* exponentials were modulated to 10 and 26, respectively. For the designed virtual compounds **3**–**11** (see Supporting Table S1 and Section 3 for further details), the WTE bias was reweighted alternatively combining Dih_cor_ and H_bonds_ with an additional CV: the root-mean-square distances (RMSD) from the coordinates of the backbone heavy atoms of the *i*RGD lowest-energy conformation predicted by PT-WTE.

The GROMACS 2018.8^58^ code patched with the PLUMED 2.5.6 plugin^59,60^ was used to run the PT-WTE simulations. The peptide was parametrized using the ff14SB Amber force field^61^ and then solvated in a 12.0 Å layer rhombic dodecahedron box using the TIP3P water model parameters.^62^ Prior to metadynamics, each replica of each system was equilibrated through 5 ns of MD under NPT conditions at 1 atm and 300 K. A time step of 2 fs in a leapfrog integrator was used. All covalent bonds were constrained to their equilibrium value using the LINCS algorithm.^63^ The Lennard–Jones potential was used to compute atom–pair interactions, with a cutoff of 10.0 Å. The simulations were carried out in periodic boundary conditions using the particle mesh Ewald (PME) to treat long-range electrostatic (grid spacing = 1.0 Å) interactions. The clustering of the peptides’ conformations corresponding to the various free energy minima was performed with the gmx cluster tool. Specifically, the clustering was performed with the GROMOS algorithm by considering the RMSD of the peptide cyclic backbone atoms (including the disulfide bridge) using a cutoff of 1.5 Å.

### 2.3 Convergence and Error Analysis

The convergence of the PT-WTE calculations was carefully assessed in both quantitative and qualitative ways. As for compound **1**, the computation of the FES at regular time intervals (Figure S1A) highlighted that after the first 80 ns (per replica) of simulations, the overall shape of the free energy landscape is conserved. Then, a block averaging analysis estimated the error associated with the Δ*G* computation (Figure S1B) at the acceptable value of ≈1 kJ/mol. In parallel, the convergence of the parallel tempering scheme was also evaluated. To further prove the reliability of our results, we monitored the values of the CVs (Figure S1C) employed for reweighting in the continuous (demuxed) trajectories of each replica. The plots in Figure S1C show a diffusive behavior of both CVs in all of the replicas of compound **1**, suggesting that no simulation was stuck in a particular region of the phase space (that is, no observed hysteresis). The average exchange acceptance ratio was ≈25%, which testifies to a good diffusion of all of the replicas over the entire temperature range (Figure S2).

### 2.4 Homology Modeling

Due to the lack of any experimental three-dimensional (3D) structure in the Protein Data Bank (PDB), a homology model of the αvβ5 head was built. Since the αv subunit had already been solved in the X-ray structures of the αvβ3, αvβ6, and αvβ8 isoforms,^64−68^ only the β5 subunit was actually modeled. First, a multiple sequence alignment between the heads (region corresponding to β3 residues 109–353) of all of the human RGD β subunits (β1, β3, β5, β6, β8) was performed with ClustalOmega^69,70^ (Figure S3). This analysis showed that two isoforms, namely, β3 and β6, possess the highest identity rate with β5 (65 and 58%, respectively). Unsurprisingly, most of the mutations and all of the amino acidic gaps occur at the specificity determining loop (SDL) region, which is at least two residues longer in β5 than in any other RGD integrin. Given the importance of the SDL for the ligand binding to the integrins orthosteric site, particular attention was paid to the modeling of this portion. Therefore, a further sequence alignment restricted to the SDL region was performed (Figure S4). In this comparison, β6 showed a higher (44%) local (SDL region) identity value than β3 (41%); thus, the crystal structure of αvβ6 in complex with the LAP peptide of TGF-β (PDB code: 4UM9(66)) was chosen as a template.^66^ Then, the knowledge-based method implemented in Prime^71^ was used to build the 3D receptor model. Furthermore, a refinement was carried out for loops bearing amino acids with missing coordinates (i.e., not coming from the template) by means of the Maestro “Refine Loops” panel.^72^ Specifically, short loops were refined using default sampling rates, whereas the folding of the SDL residues comprised between the conserved disulfide bridge C176–C185 was refined using the Extended protocol implemented in Prime.^71^ Finally, the coordinates of all of the nonconserved side chains were optimized using an energy cutoff of 10 kcal/mol. For the numbering of the β5 and β6 residues over the text, we followed the common practice to align both the receptors to a reference, represented by the first-published αvβ3 crystal structure (PDB code: 1L5G(64)). The resulting model was validated by computing the protein Ramachandran plot (Figure S5A) with the aid of the MolProbity Web server (http://molprobity.biochem.duke.edu) and evaluating the stability of the secondary structure elements in a 2 μs long MD simulation (Figure S5B).

### 2.5 Molecular Docking

Docking of the PT-WTE-predicted conformation of **1** was performed in the integrin head of αvβ5 (homology model) as well as of αvβ3 and αvβ6 (crystal structures: 4MMX(65) and 4UM9,^66^ respectively). Both the ligand and the receptors were prepared using the Protein Preparation Wizard tool, implemented in the Maestro Suite 2019.^73^ The cocrystallized Mg^2+^ and Ca^2+^ divalent cations at the protein “metal ion-dependent adhesion site” (MIDAS), “adjacent to MIDAS” (ADMIDAS), and “ligand-associated metal ion-binding site” (LIMBS) were retained and treated using the default parameters. Correct bond orders were assigned, missing hydrogen atoms were added, and all of the water molecules were deleted from the receptor structure. Then, protonation and tautomeric states at pH 7.4 were assigned to the side chains using Epik.^74,75^ Finally, the positions of all of the hydrogens were minimized. A virtual box of 30 Å × 30 Å × 30 Å, surrounding the typical RGD binding site, was selected as the search area by the means of the Receptor Grid Generator tool of Glide 8.5.^76,77^ Docking calculations were performed employing the Glide SP-peptide protocol and the OPLS3A force field.^78^ The peptide backbone was kept fixed in order to preserve the conformation obtained from the PT-WTE simulation, while all of the other parameters were kept to default values. Thus, the obtained solutions were clustered based on the ligand RMSD (default parameters) and ranked according to the Glide SP scoring function.^76,77^ The designed virtual compounds **4**, **6**, **8**, and **11** (see Supporting Table S1 and Section 3) were docked in the energy-minimized averaged MD structure of αvβ6 using the same protocol. In this case, however, positional restraints were used to discard all of the solutions, not respecting the typical RGD binding pattern.

### 2.6 Molecular Dynamics

All of the proteins and the peptide were parametrized using the ff14SB Amber force field.^61^ The divalent cations present in the integrins structures were treated with the parameters developed by Panteva et al.^79^ The PMEMD engine (GPU version) of AMBER 18^80^ was used to perform the simulations. The short-range interactions were defined as all possible contacts within a cutoff of 10 Å from every simulated atom. The long-range electrostatic interactions were computed through the particle mesh Ewald method^81^ using a 1.0 Å grid spacing in periodic boundary conditions. The iterative SHAKE algorithm^82^ was applied to constraint all bonds containing hydrogens, allowing for a 2 fs integration time step. In order to solve all of the steric clashes, each system underwent 30,000 steps of mixed steepest descent/conjugated gradient energy minimization. Then, each complex was equilibrated and heated up to 300 K, alternating NPT and NVT cycles (125,000 steps each) with the Langevin coupling bath^83^ and the Berendsen barostat,^84^ while applying gradually decreasing harmonic constraints on the heavy atoms of the protein and ligand. Finally, a production run of 2 μs was performed for each ligand–protein complex in the NPT ensemble with a target pressure and temperature of 1 atm and 300 K, respectively.

## 3 Results and Discussion

### 3.1 In Vitro Binding Assay

To provide a more exhaustive picture of the integrin affinity of *i*RGD, direct solid-phase binding assays were performed between **1** and its known target receptors αvβ3 and αvβ5 as well as the clinically important αvβ6 isoform (Table 1 and see Section 2 for details).

**Table 1:** Binding Affinity of **1 t**oward αvβ3, αvβ5, and αvβ6

	IC_50_ (nM)^a^		
compound	αvβ3	αvβ5	αvβ6
**1** (*i*RGD)	36 ± 14	75 ± 10	191 ± 44
cilengitide^b^	0.84 ± 0.21	2.4 ± 0.5	n.t.^d^
**2** ([RGD-Chg-E]-CONH_2_^c^)	n.t.^d^	n.t.^d^	1.1 ± 0.2

a Data are shown as the mean of three independent experiments ± SEM.

b Cilengitide^49,50^ was used as an internal reference compound for both αvβ3 and αvβ5.

c [RGD-Chg-E]-CONH_2_^51^ was used as an internal reference compound for αvβ6.

d Not tested.

In line with the previously reported data,^16^*i*RGD showed a mid-low nanomolar potency toward both αvβ3 and αvβ5. More interestingly, we here detected for the first time the peptide’s affinity for the αvβ6 isoform, albeit with an IC_50_ higher than those measured for the αvβ3 and αvβ5 subtypes. The renewed integrin binding profile of **1** introduces intriguing questions and new perspectives. In particular, what is the molecular basis for this specificity? Can *i*RGD be modified to selectively shift its affinity toward each of the three isoforms? In an attempt to answer these queries, we carried out a full characterization of the *i*RGD binding mechanism. In the first stage, we investigated the intrinsic folding properties of the peptide, leading to the identification of its in-solution conformation.

### 3.2 Folding of *i*RGD

**Figure 1:**
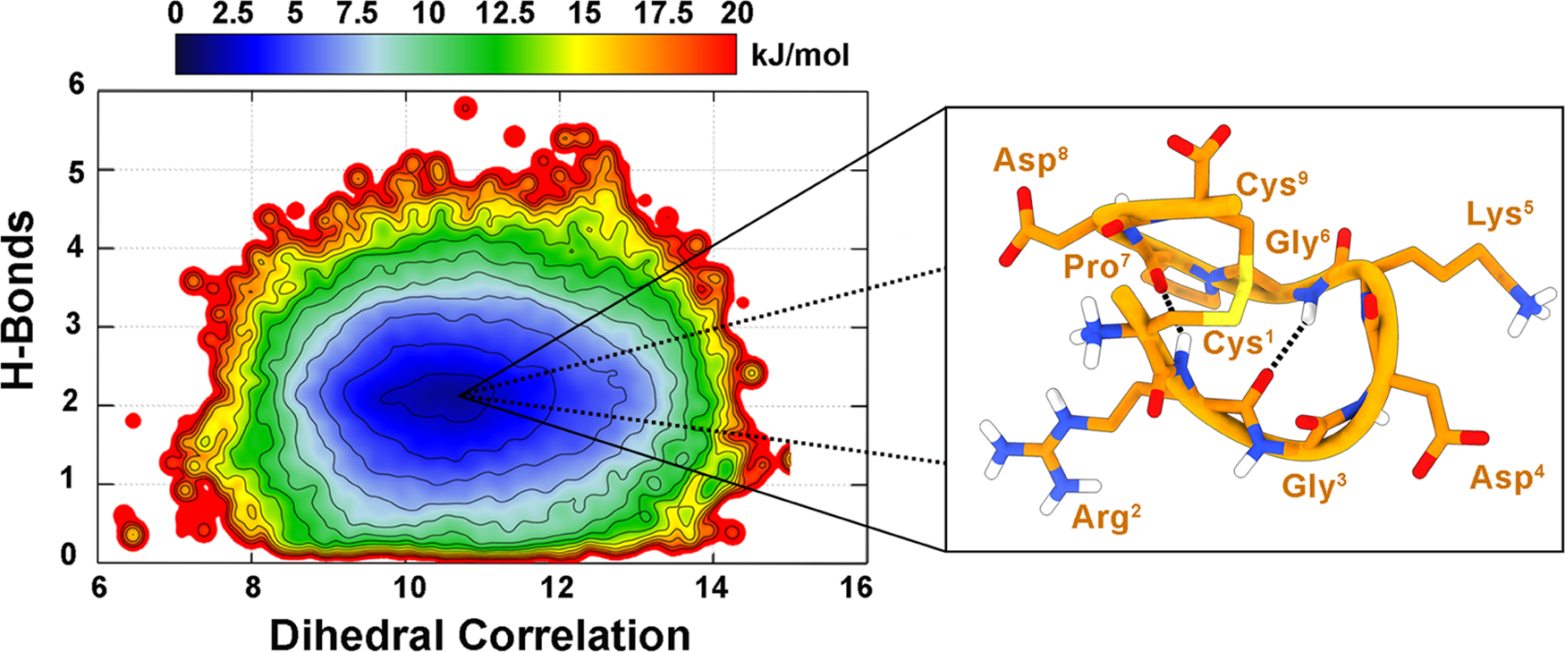
Free energy surface (FES) of the folding process of **1** as a function of Dih_cor_ and H_bond_ CVs with isosurfaces displayed every 1.5 kJ/mol. The conformation representing the main free energy minimum is shown as the inset.

Peptide and protein folding are events that naturally occur in time scales (tens of μs to ms) not accessible to standard molecular simulations. For this reason, we adopted an enhanced sampling approach, namely, PT-WTE metadynamics, to study the conformational behavior of *i*RGD in water. PT-WTE is a theoretical method widely employed for predictions of small proteins’ and peptide’s folding,^85−93^ which requires no prior knowledge of the system under study: the sampling is boosted by the combination of a typical parallel tempering scheme with a metadynamics (MetaD) bias potential deposited on the potential energy of the system (WTE ensemble). Once the simulation is converged, the user can define some collective variables (CVs) to compute the free energy surface (FES) through the desired reweighting scheme. In our case, 6 parallel replicas were employed to span the temperature range going from 300 to 450 K in the WTE ensemble. Each replica was simulated for 140 ns for a total simulation time of 840 ns, in which the conformational space of the peptide was widely explored (Figure S1C). At the end of the calculation, the MetaD bias of the 300 K replica was reweighted according to the Tiwary–Parrinello^57^ algorithm. Indeed, the FES was computed as a function of two CVs specifically selected for describing the folding (see Section 2 for details): (i) the degree of similarity between contiguous dihedral angles of the backbone (dihedral correlation, Dih_cor_), which can help to describe backbone conformational changes^94^ and (ii) the number of intramolecular backbone–backbone hydrogen bonds (H_bonds_) that can be indicative of the presence of specific secondary structure elements. Looking at the resulting FES (Figure 1), a single energy minimum can be identified. The structures contained in this energy basin were clustered based on the RMSD of all of the backbone heavy atoms, highlighting the presence of a unique predominant conformation (>90% of occurrence). In the latter, **1** is folded in a peculiar horseshoe-like shape, characterized by two hydrogen bonds formed by (i) the carbonyl oxygen of Arg^2^ with the amide nitrogen of Gly^6^ and (ii) the carbonyl group of Pro^7^ with the amide nitrogen of Arg^2^. The reliability of this result is strongly supported by the high convergence reached by the calculation and the low computed error (≈1 kJ/mol) associated with the Δ*G* prediction (Figure S1 and Section 2 for details). The identification of the low-energy horseshoe-like conformation of *i*RGD represents an important achievement, as it is known that conformations of peptides in aqueous environments often overlap with the receptor-bound one.^51,95,96^

### 3.3 Binding Mode Studies

Starting from the PT-WTE results, extensive computational studies were carried out to unravel the binding modalities of *i*RGD toward αvβ3 and αvβ5 as well as the newly discovered biological partner αvβ6. An initial guess of the ligand binding poses was obtained by performing molecular docking in the X-ray structures of αvβ3 and αvβ6, as well as in the newly built αvβ5 homology model (see Section 2 for details). The PT-WTE-predicted conformation of *i*RGD was used as the starting point for the docking calculations. According to the employed protocol, the macrocyclic backbone of **1** was kept rigid, while sampling of the side chains’ orientation was allowed. Docking in both αvβ3 and αvβ5 converged toward a well-defined binding pose (Figure S6A,B) in which **1** adopts the typical RGD binding pattern. In particular, the peptide’s Arg^2^ forms a salt bridge with the conserved (αv)-D218 and a cation–π interaction with (αv)-Y178, whereas the carboxylate group of Asp^4^ chelates the Mg^2+^ cation at the protein MIDAS (‘metal ion-dependent adhesion site’) in the β subunit. Notably, the most relevant difference between the two docking poses consists in the position of the peptide’s residues flanking the RGD motif (a.a. 5–9) with respect to the region defined by the specificity determining loop (SDL) that is distinctive of the various integrin subtypes.^51,96−99^ Indeed, in αvβ3, the flanking amino acids of **1** approximate this region, while in αvβ5, they are predicted to point in the opposite direction, establishing two H-bonds with the side chains of (β5)-T315 and (β5)-N317.

**Figure 2:**
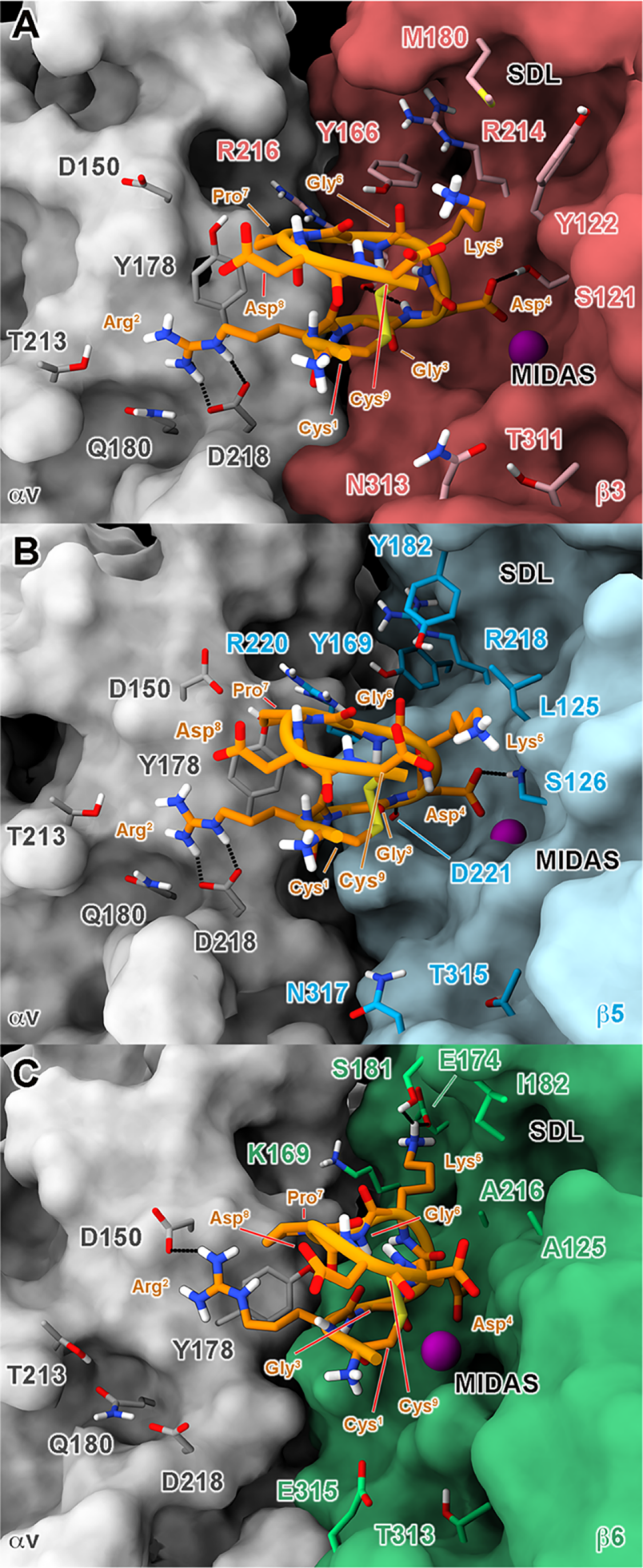
MD-predicted binding mode of *i*RGD at the orthosteric binding site of (A) αvβ3, (B) αvβ5, and (C) αvβ6 integrins. The different receptor subunits are depicted as colored surfaces (αv = gray, β3 = red, β5 = cyan, and β6 = green). Amino acids important for peptide binding are highlighted as sticks, while the Mg^2+^ ion in MIDAS is shown as a purple sphere. The ligand is represented as orange ribbons and sticks; nonpolar hydrogens are omitted for the sake of clarity; and H-bonds are shown as black dashed lines.

**Figure 3:**
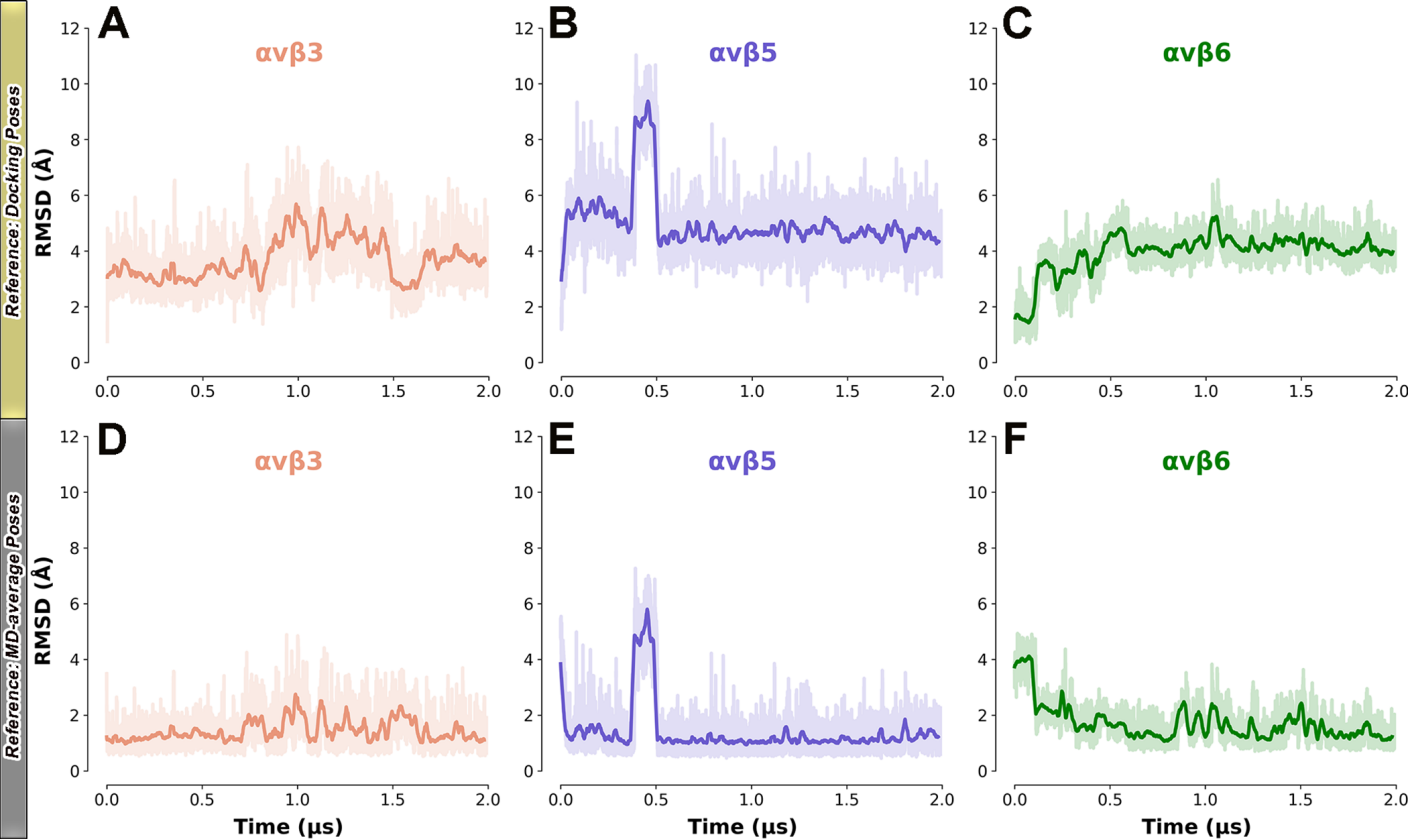
Ligand’s RMSD plots for the three simulated binding complexes. Prior to the RMSD computation, the trajectories were aligned on the Cα-atoms of the most stable secondary structure elements. Two different ligand’s reference conformations were used: (i) the first frame (equilibrated docking pose) of each MD complex (first row: A–C) and (ii) the average binding pose observed during each MD run (second row: D–F). The RMSD values (bolded lines) are smoothed with a rolling window of 5 ns, while the actual fluctuations are shown with a slight transparency.

**Figure 4:**
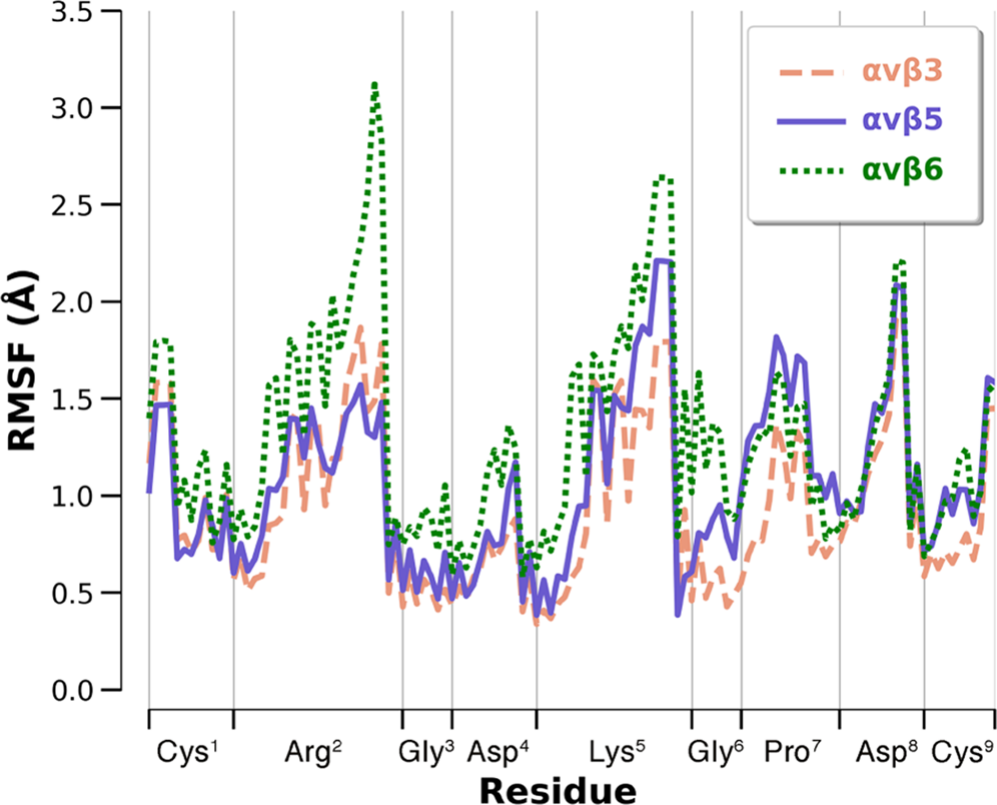
*i*RGD (**1**) residues RMSF in three binding complexes. The computation was performed on all of the ligand’s heavy atoms.

The meaning of such small differences together with the energetics and the overall stability of the docking complexes was then evaluated in 2 μs long MD simulations. A refinement of the binding poses was indeed desirable to optimize potential clashes or small artifacts due to the use of both a rigid receptor and restraints on the ligand’s peptide backbone upon docking. In this perspective, long MD trajectories can allow the system to escape from relative energy minima in which it might be trapped, fully considering the receptor flexibility, the solvent effects, and the entropic contributions, which are neglected during docking calculations. The MD results showed that, in both αvβ3 and αvβ5, *i*RGD slightly rearranges during the first 20–100 ns of the simulation to assume a binding mode that is mostly conserved for the rest of the trajectory (Figure 2). This trend is confirmed by the ligand’s RMSD with respect to either the first frame (Figure 3A,B) or the peptide’s average position in each MD trajectory (Figure 3D,E). Notably, the ligand’s rearrangement is more evident in the *i*RGD/αvβ5 complex, where **1** loses the interactions with (β5)-T315 and (β5)-N317 predicted by docking (Figure S7) to get closer to the SDL pocket (Figure S8) as in αvβ3. The overall stability of the final peptide binding conformation was further assessed by the analysis of the ligand’s root-mean-square fluctuation (RMSF), showing very low per-residue fluctuations over the 2 μs time scale (Figure 4). A detailed analysis of the ligand–receptor interactions throughout the MD trajectories was thus performed. First, it was shown that the typical interaction scheme involving the RGD motif is highly conserved over time in both αvβ3 and αvβ5 (Figures S9 and S10). Then, we observed that during the MD runs, new contacts are established by **1** in each of the investigated complexes. In detail, the Asp^4^ side chain and the amide backbone form hydrogen bonds with (β3)-S121 and (β3)-R216 in αvβ3 and with (β5)-S126 and (β5)-D221 in αvβ5. Additionally, van der Waals interactions are formed by the peptide Pro^7^ and the aromatic ring of (αv)-Y178. Finally, in the *i*RGD/αvβ3 complex, a transient H-bond is detected between the backbone of the ligand’s Lys^6^ with the side chains of (β3)-R214 (Figure S9). It is also interesting to note that the highly compact folding assumed by the peptide is conserved throughout the simulations. In this regard, we report that very low ligand’s backbone RMSD values were computed with respect to the horseshoe-like conformation predicted by PT-WTE, especially in the most affine receptor αvβ3, and that the two stabilizing intramolecular H-bonds are retained in more than 90% of both the entire trajectories (Figures S11 and S12). These outcomes are further indicative of the similarity between the in-solution and receptor-bound conformations of *i*RGD.

As concerns the αvβ6 receptor, the initial docking mode was generally similar to those observed in αvβ3 and αvβ5. (Figure S6C). However, during the following MD simulation, the peptide’s pose as well as its overall folding was less conserved than those in the other two investigated subtypes, as testified by the ligand’s RMSD (Figure 3) and RMSF (Figure 4). The latter shows that especially the RGD motif has higher fluctuations than observed in αvβ3 and αvβ5, suggesting a lower stability of the typical RGD interactions.

In detail, a certain tendency of Arg^2^ to break its ionic contact with (αv)-D218 and to bind, in turn, the side chain of (αv)-D150 (Figure S13) is observed; furthermore, the Mg^2+^ chelation is not fully conserved over the simulation (Figure S14), as also testified by the higher changes in the Gly^2^-ψ and Asp^3^-φ dihedral angles with respect to the MD trajectories on αvβ3 and αvβ5 (Figure S15).

Likewise, even the backbone of **1** is more fluctuating than in the other two systems, as shown by the RMSD plots computed against the predicted PT-WTE conformation (Figure S16). These observations, along with the general trend observed in the three simulations, agree with the selectivity profile of the in vitro assays and with the lower binding affinity of **1** for αvβ6 with respect to αvβ3 and αvβ5.

Notably, our in silico predictions are coherent with both the bioactivation mechanism and the chemical functionalization of *i*RGD. Indeed, in the MD-refined complexes, both the ligand’s Lys^5^ and Gly^6^ residues and the terminal Cys^9^ carboxylate group are solvent-exposed; moreover, the latter is not involved in any specific interaction with the receptor. Thus, the Lys^5^–Gly^6^ bond is easily accessible for proteolytic cleavage, which releases the NRP-1-recognizing CendR/K sequence (CRGDK). On the other hand, the solvent exposure of the Cys^9^ carboxylate explains why the bioconjugation of this group with bulky molecules, for either therapeutic or diagnostic purposes, is safely permitted.^18−19,20,25,26,35^ Both of these findings strengthen the reliability and increase the scientific impact of the obtained atomistic *i*RGD–integrin binding complexes.

### 3.4 Structural Basis of Integrin Selectivity and Hints for Drug Design

The range of theranostic applications of RGD integrin ligands highly depends on their activity and selectivity profiles. For this reason, the development of potent and subtype-specific compounds is a desirable, albeit challenging, task.^100,101^ In this perspective, the presented interaction models can help to rationalize the molecular basis of the selectivity of **1** for αvβ3, αvβ5, and αvβ6 and, in turn, provide hints to modulate the affinity toward each single isoform. In fact, the MD-predicted complexes suggest that the trend observed in the experimental binding assays can be ascribed to the punctiform mutations occurring in the SDL cavities (Figure S17) of the three receptors. These, in turn, cause changes in the steric and electrostatic requirements for the binding of RGD-featured ligands. As previously reported,^51,96−99^ the core of the (β3)/(β5)-SDL regions is occupied by the bulky side chains of (β3)-Y166/(β5)-Y169 (β2-β3 loop), (β3)-R214/(β5)-R218, and (β3)-R216/(β5)-R220 (α2-α3 loop), which are kept in place by a π–cation network where the tyrosine ring is interposed between the guanidinium groups of the two arginines (Figure S18 A,B). For this reason, high potency toward αvβ3 and αvβ5 is obtained by ligands able to orient the residues flanking their RGD motif in opposite direction to the bulky SDL pockets of these integrins.^64,102^ A prominent example is the most famous αvβ3/αvβ5 dual modulator cilengitide,^49,50^ in which the residues next to the RGD motif (d-Phe^4^ and *N*Me-Val^5^) face away from the α/β protein interface (Figure S19). Similarly, **1** avoids steric clashes with the hindered SDL cavities of αvβ3 and αvβ5, thus retaining the affinity for both receptors. This is allowed by the compact bound conformation assumed by *i*RGD in the two isoforms, as predicted by our MD simulations. Indeed, the peptide exposes toward the SDL region the small Gly^6^ and the flexible Lys^5^, whose side chain assumes an orientation closely resembling that of the cilengitide’s phenyl ring (Figure S19). Nonetheless, the potency of *i*RGD toward both these integrins could be further improved by increasing the number of interactions played with the αv subunit. For instance, our binding models suggest that the substitution of standard Pro^5^ with hydroxy-functionalized forms of the same amino acid, such as 4- or 5-hydroxyproline, could allow the compound to form an additional H-bond with (αv)-Y178.

On the other hand, achieving αvβ3/αvβ5 selectivity is far from being trivial due to the very similar steric and electrostatic features of the SDL region in the two receptors. Indeed, to our knowledge, most of the known medium-sized peptides targeting αvβ5 also retain activity toward the αvβ3 isoform.^103^ However, in the case of *i*RGD, our simulations propose that a receptor area different from the SDL could be targeted to discriminate between the αvβ3 and αvβ5 subtypes.

**Figure 5:**
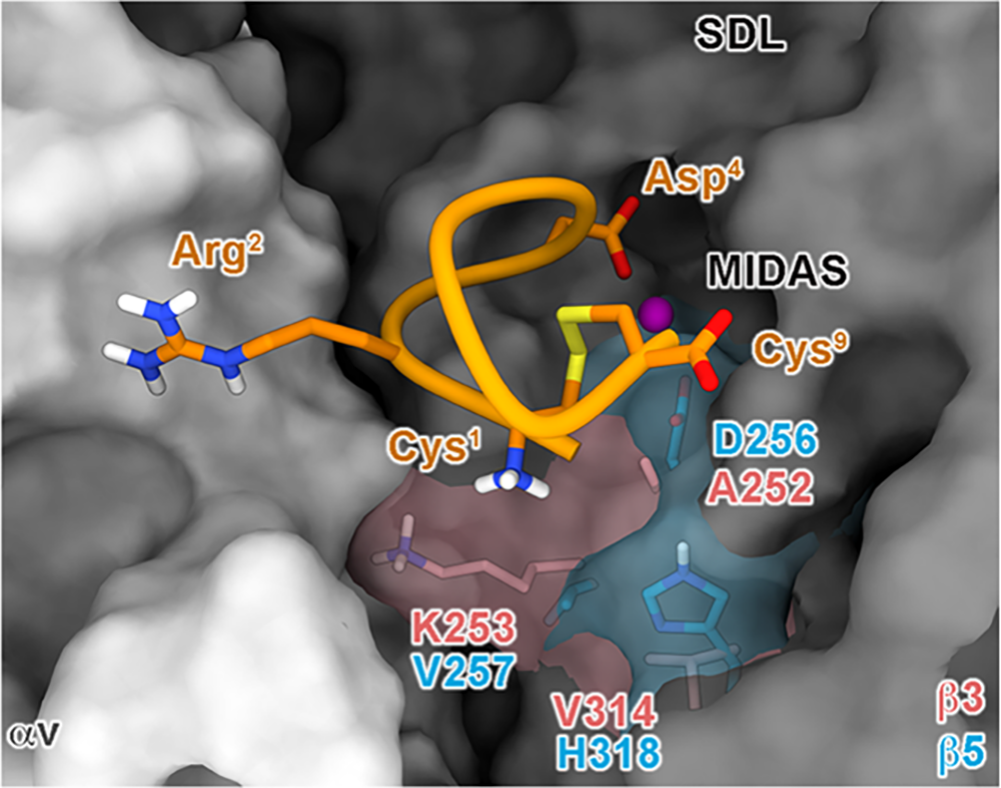
Possible hints for achieving αvβ3/αvβ5 selectivity. The N-terminus of **1** in its predicted binding modes at αvβ3/αvβ5 is close to a subpocket where three key mutations occur. The αv and β3/β5 subunits are depicted as light and dark gray surfaces, respectively. The key mutations between the two receptors are highlighted as red (β3) and cyan (β5) sticks. The ligand is represented as orange ribbons and sticks; nonpolar hydrogens are omitted for the sake of clarity.

Particularly, the N-terminal amine group of **1** is directed toward the bottom part of the RGD binding site, where the integrins’ molecular surfaces delineate a small cleft (Figure 5). At this level, β3 and β5 are distinguished by three mutations, namely, (β3)-A252/(β5)-D256, (β3)-K253/(β5)-V257, and (β3)-V314/(β5)-H318, which could be taken into account to apply isoform-specific chemical modifications at the peptide’s N-terminus.

In this regard, recent studies have shown that the functionalization of this moiety can be safely performed without affecting the *i*RGD biological properties. Indeed, a variant of the peptide in which the Cys^1^ N-terminus and the Cys^9^ C-terminus are capped with acetyl and *N-*methyl groups, respectively, has been recently developed, showing striking tumor-homing and -penetrating features.^104^

**Figure 6:**
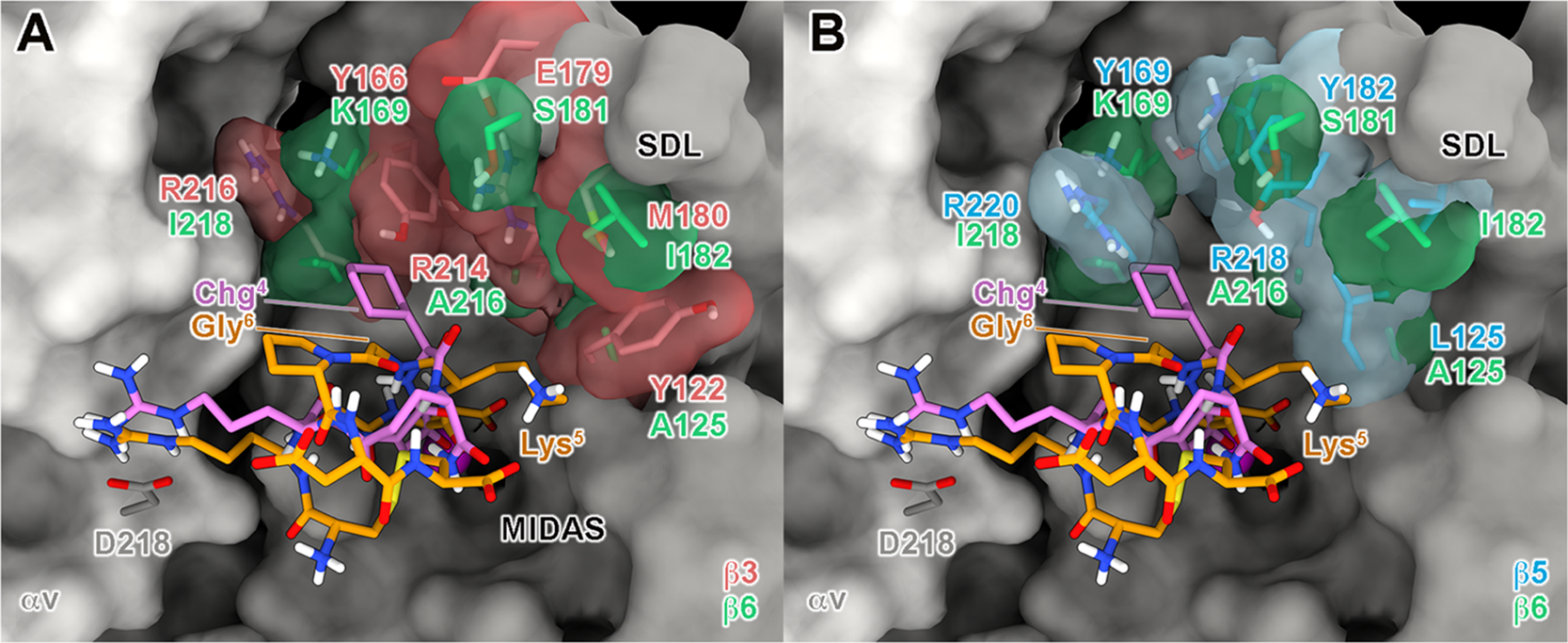
Superposition of the MD-predicted binding pose of *i*RGD at αvβ3 (A) and αvβ5 (B) with that of [RGD-Chg-E]-CONH_2_ at αvβ6. The αv and β subunits are depicted as light and dark gray surfaces, respectively. The key mutations between the three receptors are highlighted as red (β3), cyan (β5), and green (β6) sticks, contoured by transparent surfaces. Peptides **1** and **2** are depicted as orange and magenta sticks, respectively; nonpolar hydrogens are omitted for the sake of clarity.

At variance with the β3 and β5 subunits, β6 displays a wider and more lipophilic SDL cavity. Here, (β3)-R214/(β5)-R218 and (β3)-R216/(β5)-R220 (α2-α3 loop) are replaced, respectively, by the smaller (β6)-A216 and by the hydrophobic (β6)-I218, while (β3)-Y66/(β5)-Y169 is mutated in (β6)-K169 (Figures 6 and S18C). Notably, in our simulation on the **1**–αvβ6 complex, this residue is engaged in stable salt bridges with the carboxylic groups of (αv)-E121 and (αv)-D123; these interactions attract its side chain toward the αv subunit, further increasing the ligand-accessible volume within the SDL cavity (Figure S18C). In this scenario, *i*RGD is prompted to rearrange, as testified by the ligand’s RMSD evolution (Figure 3C,F), and occupy the cleft forming H-bonds with (β6)-E174 and (β6)-S181 through its Lys^5^ side chain (Figures 2 and S13). These movements can partially impair both the peptide’s folding (Figure S16) and the interaction scheme of the RGD motif (Figures S13–S15), contributing to explaining the lower affinity of **1** for αvβ6. In fact, higher potency toward this integrin is displayed by peptides that occupy the SDL through bulky lipophilic moieties while still preserving the correct RGD binding pattern. Representative examples are compounds showing a helical DLXXL/I motif like the endogenous αvβ6 ligand latency-associated peptide (LAP) of the transforming growth factor β (TGF-β)^66^ or the small selective cyclic pentapeptide developed by us, namely, [RGD-Chg-E]-CONH_2_ (**2**).^51,105^ The different αvβ6 interactions between these peptides and *i*RGD are highlighted by the superposition of the binding pose of **1** and **2** in this integrin receptor (Figure 6), which provides potential clues for the rational modification of the peptide. A possible strategy to selectively increase the *i*RGD’s affinity for αvβ6 might thus consist of the insertion of properly oriented bulky moieties able to target the wide and lipophilic SDL region of this integrin while being not tolerated by both the αvβ3 and αvβ5 isoforms. In this regard, the comparison of our interaction model to the 3D complex of αvβ6 and our selective pentapeptide **2** (Figure 6) shows that the Lys^5^ and Gly^6^ of *i*RGD partly overlap with the cyclohexylglycine (Chg^4^) of the reference compound, being prone to interact with the SDL groove.

**Figure 7:**
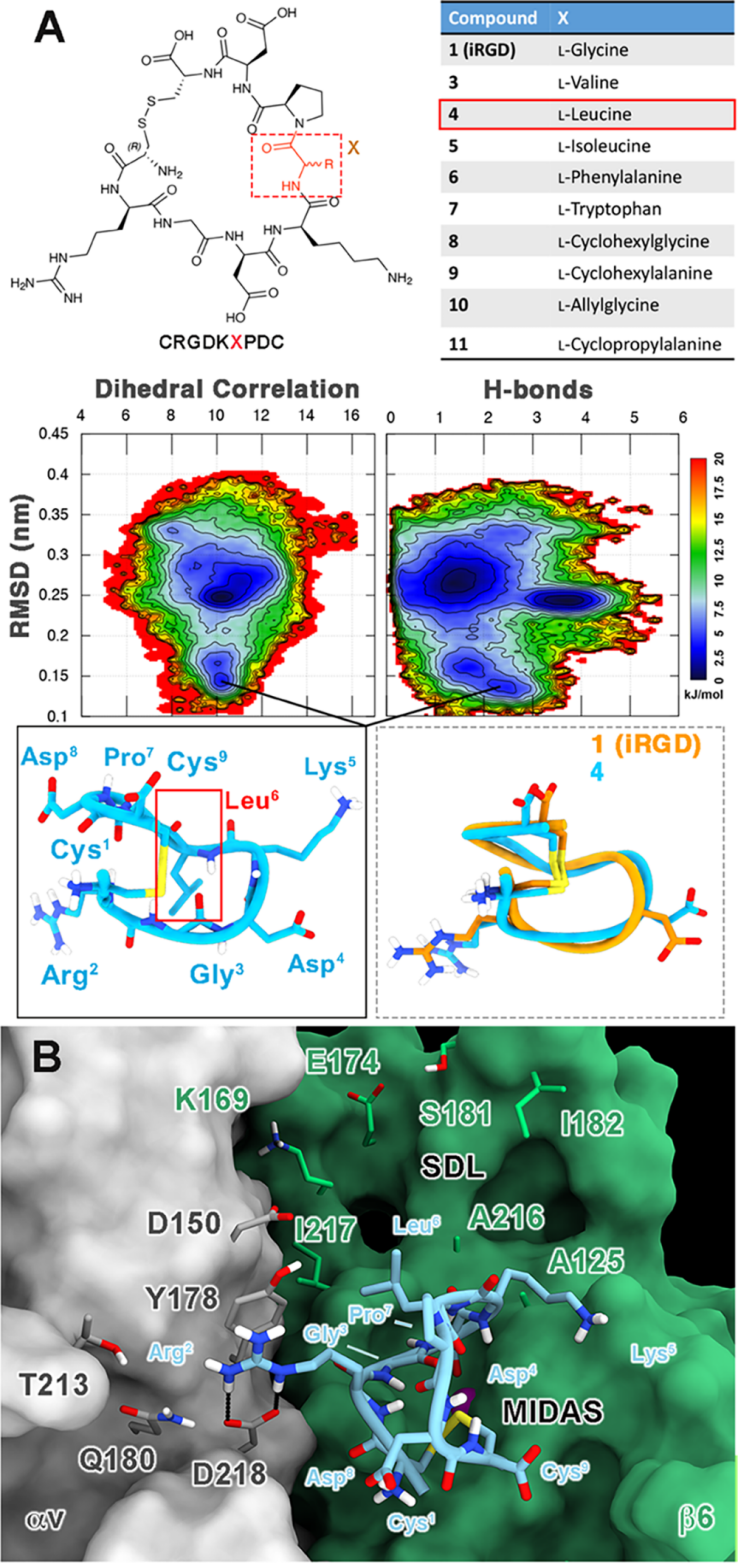
(A) Hints for increasing αvβ6 affinity and selectivity. A virtual library of nine compounds was designed by mutating Gly^6^ of **1** with natural and non-natural lipophilic amino acids (upper panel). Free energy surfaces (FESs) of the folding process of **4** with isosurfaces were displayed every 1.5 kJ/mol (lower panel). The horseshoe-like conformation of **4** and its superimposition with **1** are shown as insets. (B) Docking-predicted binding mode of compound **4** at the RGD binding site of the αvβ6 integrin. The different receptor subunits are depicted as colored surfaces (αv = gray and β6 = green). Amino acids important for peptide binding are highlighted as sticks, while the Mg^2+^ ion in the MIDAS is shown as a purple sphere. The ligand is represented as cyan ribbons and sticks; nonpolar hydrogens are omitted for the sake of clarity; and H-bonds are shown as black dashed lines.

Since Lys^5^ is part of the CendR sequence (needed for the interaction with NRP-1), it could be replaced only by amino acids sharing analogous physicochemical properties (i.e., arginine). Therefore, a focused chemical optimization campaign could be performed by replacing Gly^6^ with natural (i.e., valine, leucine, isoleucine, phenylalanine, tryptophan) or non-natural (i.e., cyclohexylglycine, cyclohexylalanine, cyclopropylalanine, allylglycine) lipophilic residues with moderate bulkiness so as to fill the peculiar (more hydrophobic and wider with respect to αvβ3 and αvβ5) pocket mostly made up by the αvβ6 SDL. However, given the higher flexibility of glycine with respect to all of the other amino acids, the impact of such modifications on the peptide’s folding and ability to interact with the receptor should be carefully pre-evaluated. To this aim, we designed a small virtual library of nine designed *i*RGD derivatives, whose folding properties were predicted through additional PT-WTE calculations (compounds **3**–**11**; see Table S1 and Figures 7A, S20, and S21). In particular, we investigated the capability of these compounds to adopt a conformation similar to that of **1** by computing their folding free energy surfaces as a function of two different sets of CVs.

Specifically, each of the CVs used to characterize the folding energy landscape of **1** (Dih_cor_ and H_bond_) was alternatively combined with the peptides’ RMSD values computed with respect to the horseshoe-like conformation of *i*RGD. The predicted FES showed that five out of the designed peptides (**3**, **5**, **7**, **9**, and **10**) have folding profiles quite diverging from the parent peptide (Figures S20 and S21), which may lead to a total loss or a reduction of integrin affinities. Nonetheless, the remaining four molecules (**4**, **6**, **8**, and **11**) could be able to assume a conformation comparable to that of **1**. In fact, although these peptides seem to have a more complex conformational ensemble than *i*RGD, their folding FESs present a clear energy basin at low RMSD values (1.2–1.7 Å) with respect to the horseshoe-like shape of **1**. This is particularly evident for **4**, where Gly^6^ is replaced by a leucine, showing a low-energy conformation that almost perfectly overlaps with *i*RGD (Figure 7A). To verify whether these peptides could fit into the wide and lipophilic SDL pocket of αvβ6, we performed additional semiflexible docking calculations on this receptor. Particularly, the docking pose of **4** shown in Figure 7B is representative of how the new lipophilic residue at the 6 position—here Leu^6^—could be oriented toward the hydrophobic SDL cavity and may thus contribute to increasing the αvβ6 affinity and specificity.

## 4 Conclusions

Over the past decade, the tumor-homing *i*RGD (**1**) peptide has emerged as a powerful tool for anticancer therapy,^1,17,21,25−27^ due to its high tropism for cancer tissues and the possible coadministration with a plethora of chemotherapeutic agents. In this article, we provide unprecedented data about the biological and structural aspects of *i*RGD’s mechanism of action. First, we discovered that **1** has affinity not only for the known targets αvβ3/αvβ5 integrins but also for the αvβ6 subtype. These data have great clinical relevance considering that the latter receptor is overexpressed in many malignancies, including pancreatic ductal adenocarcinoma, against which *i*RGD has shown to be a promising therapeutic weapon in combination with paclitaxel and gemcitabine.^34,37^ Second, by means of advanced metadynamics simulations, we discovered that in an aqueous environment, **1** mainly adopted a peculiar horseshoe-like conformation, which was then employed as a starting point for accurate interaction studies with the three integrin receptors through molecular dynamics simulations. Notably, our in silico binding predictions are in agreement with the peptide’s activation mechanism, relying on the proteolytical cleavage of its Lys^5^–Gly^6^ bond when bound to the integrin surface and the following release of the internalizing CendR motif. Moreover, they allowed us to rationalize at the atomic level the experimentally measured potency and selectivity trend of **1** (αvβ3 ≥ αvβ5 > αvβ6). In particular, the compact horseshoe-like shape allows the peptide to fit the peculiar features of the integrins’ SDL cleft and seems thus fundamental to achieving nanomolar binding affinity. This requirement is better satisfied in the sterically hindered αvβ3/αvβ5 integrins than in αvβ6, where this cleft is wider and lipophilic due to the presence of specific mutations that were clearly described over text. On this basis, we set up a few possible strategies to fine-tune the affinity/selectivity of *i*RGD toward each of the investigated integrins, especially designing new peptides potentially able to selectively recognize the newly emerged anticancer target αvβ6. We are confident that these clues can pave the way for a new successful drug design campaign aimed at producing *i*RGD derivatives with optimized and fine-tuned pharmacodynamic properties.

## Data Availability

All of the input files and trajectory data sets are published on a public Zenodo folder and freely available at the following link: https://zenodo.org/record/8089457.

## Supporting Information Available

PT-WTE convergence assessment and additional analysis on all of the simulated systems (PDF)

## Abbreviations

DSG2: desmoglein 2
ECM: extracellular matrix components
IPF: idiopathic pulmonary fibrosis
*i*RGD: internalizing RGD
PDAC: pancreatic ductal adenocarcinoma
NRP-1: neuropilin-1
MetaD: metadynamics
PT: parallel tempering
PT-WTE: parallel tempering in the well-tempered ensemble
WTE: well-tempered ensemble
