# Supplementary material for: Molecular View on the iRGD Peptide Binding Mechanism: Implications for Integrin Activity and Selectivity Profiles
Source: J Chem Inf Model. 2023 Oct 3;63(20):6302–15. doi: 10.1021/acs.jcim.3c01071 (PMC10598797; doi:10.1021/acs.jcim.3c01071)
Supplement: Supplementary file 1 — ci3c01071_si_001.pdf [file ci3c01071_si_001.pdf]

A Molecular View on the *i*RGD Peptide Binding Mechanism: Implications for Integrin Activity and Selectivity Profile

Vincenzo Maria D'Amore,[1,‡] Greta Donati,[1,‡] Elena Lenci,[2] Beatrice Stefanie Ludwig,[3] Susanne Kossatz,[3,4] Monica Baiula,[5] Andrea Trabocchi,[2] Horst Kessler,[4] Francesco Saverio Di Leva,[1,] Luciana Marinelli[1,*]*

[1] *Department of Pharmacy, Università degli Studi di Napoli "Federico II", Via D. Montesano 49, 80131 Naples, Italy.*

[2] *Department of Chemistry "Ugo Schiff", University of Florence, via della Lastruccia 13, I-50019 Sesto Fiorentino, Florence, Italy.*

[3] *Department of Nuclear Medicine, University Hospital Klinikum Rechts der Isar and Central Institute for Translational Cancer Research (TranslaTUM), Technical University Munich, Munich, 81675, Germany*

[4] *Department of Chemistry, Institute for Advanced Study, Technical University Munich, Garching, 85748, Germany*

[5] Department of Pharmacy and Biotechnology, University of Bologna, Via Irnerio 48, 40126 Bologna, Italy

KEYWORDS: RGD Integrins, Tumor-homing peptides, Computational Chemistry, Drug Design, Metadynamics

Supporting Information

TABLE OF CONTENTS

Table S1: Primary sequence of peptides **1-11**. **p. S2**

Figure S1: Convergence of the PT-WTE simulation. **p. S3**

Figure S2: Replica exchange plots of the PT-WTE simulation. **p. S4**

Figure S3: Multiple sequence alignment of selected RGD integrins heads. **p. S5**

Figure S4: Local multiple sequence alignment of integrins SDL region. **p. S5**

Figure S5: Validation of the αvβ5 homology model. **p. S6**

Figure S6: Docking predicted binding poses. **p. S7**

Figure S7: Interatomic distances of *i*RGD with (β5)-T315 and (β5)-N317. **p. S8**

Figure S8: Upward rotation of *i*RGD during the MD simulation in complex with αvβ5. **p .S8**

Figure S9: Analysis of the *i*RGD-αvβ3 interactions. **p. S9**

Figure S10: Analysis of the *i*RGD-αvβ5 interactions. **p. S10**

Figure S11: Stability of the peptide conformation during the *i*RGD-αvβ3 MD run. **p. S11**

Figure S12: Stability of the peptide conformation during the *i*RGD-αvβ5 MD run. **p. S12**

Figure S13: Analysis of the *i*RGD-αvβ6 interactions. **p. S13**

Figure S14: RGD binding pattern assumed by *i*RGD in αvβ6. **p. S14**

Figure S15: Analysis of the *i*RGD's ϕ-Gly3 and ψ-Asp^4 torsion values during MD runs. **p. S14**

Figure S16: Stability of the peptide conformation during the *i*RGD-αvβ6 MD run. **p. S15**

Figure S17: Cartoon representation of the integrins' RGD binding site and SDL cavity. **p. S15**

Figure S18: Punctiform mutations occurring at the SDL pocket of **the** β3, β5 and β6 subunits. **p. S16**

Figure S19: Superposition between the MD poses of *i*RGD at αvβ3 and αvβ5 and cilengetide. **p. S17**

Figure S20: PT-WTE-predicted folding Free Energy Surfaces of compounds **3-7**. **p. S18**

Figure S21: PT-WTE-predicted folding Free Energy Surfaces of compounds **8-11**. **p. S19**

Table S1. Primary sequence of **1-11**. Compounds **3-11** are virtually designed peptides.

**Compound**	**Sequence**
**1 (*i*RGD)**	Cys-Arg-Gly-Asp-Lys-Gly-Pro-Asp-Cys
**2**	[Arg-Gly-Asp-Chg-Glu]-$CONH_2$
**3**	Cys-Arg-Gly-Asp-Lys-Val-Pro-Asp-Cys
**4**	Cys-Arg-Gly-Asp-Lys-Leu-Pro-Asp-Cys
**5**	Cys-Arg-Gly-Asp-Lys-Ile-Pro-Asp-Cys
**6**	Cys-Arg-Gly-Asp-Lys-Phe-Pro-Asp-Cys
**7**	Cys-Arg-Gly-Asp-Lys-Trp-Pro-Asp-Cys
**8**	Cys-Arg-Gly-Asp-Lys-Chg-Pro-Asp-Cys
**9**	Cys-Arg-Gly-Asp-Lys-Cha-Pro-Asp-Cys
**10**	Cys-Arg-Gly-Asp-Lys-Alg-Pro-Asp-Cys
**11**	Cys-Arg-Gly-Asp-Lys-Cpa-Pro-Asp-Cys

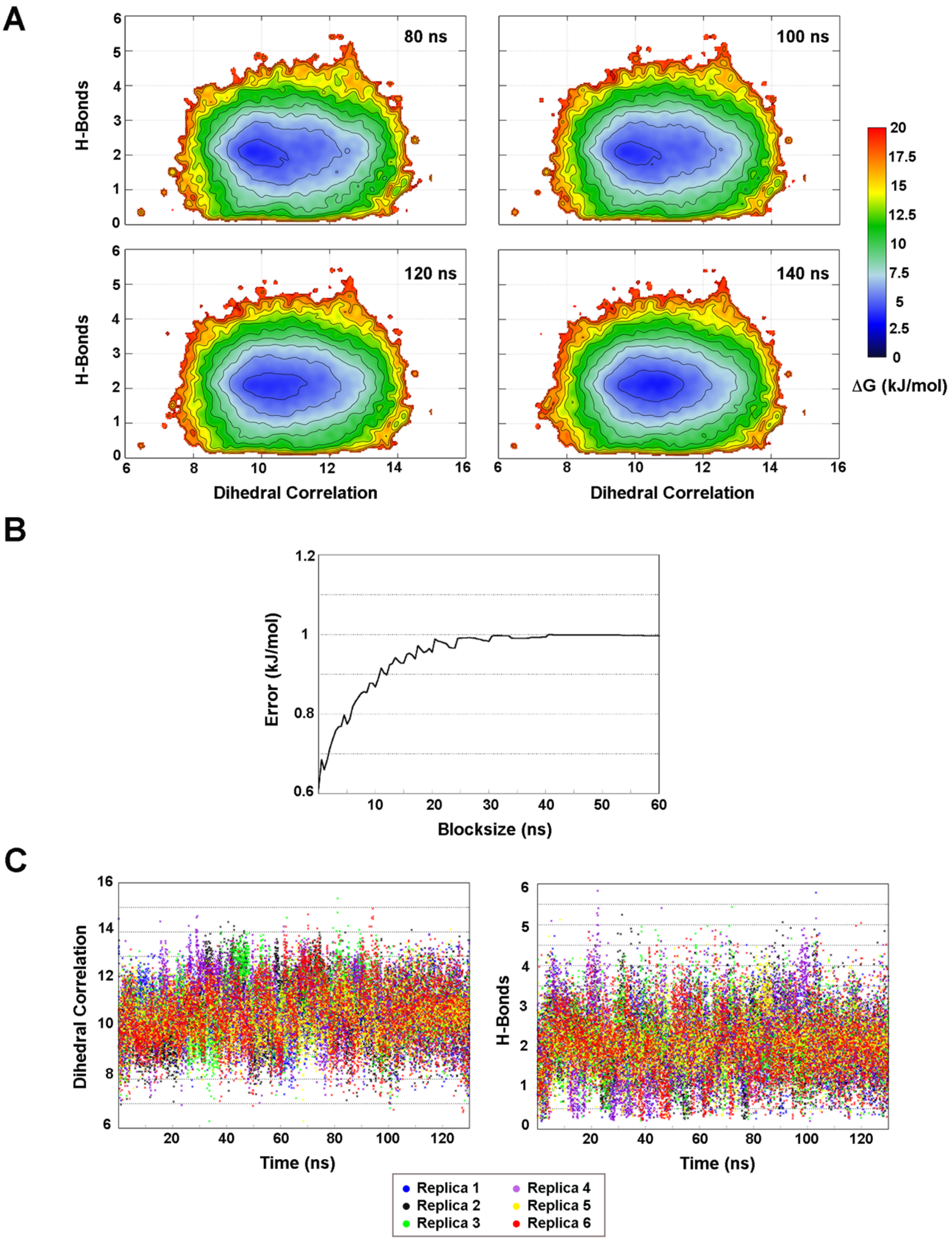

Figure S1. Convergence of PT-WTE calculation on **1**. A) Time evolution of the FES during the last 60 ns of simulation. B) Quantitative assessment of the error associated with the FES calculation trough block averages analysis. C) CVs diffusion in the six demuxed (continuous) trajectories.

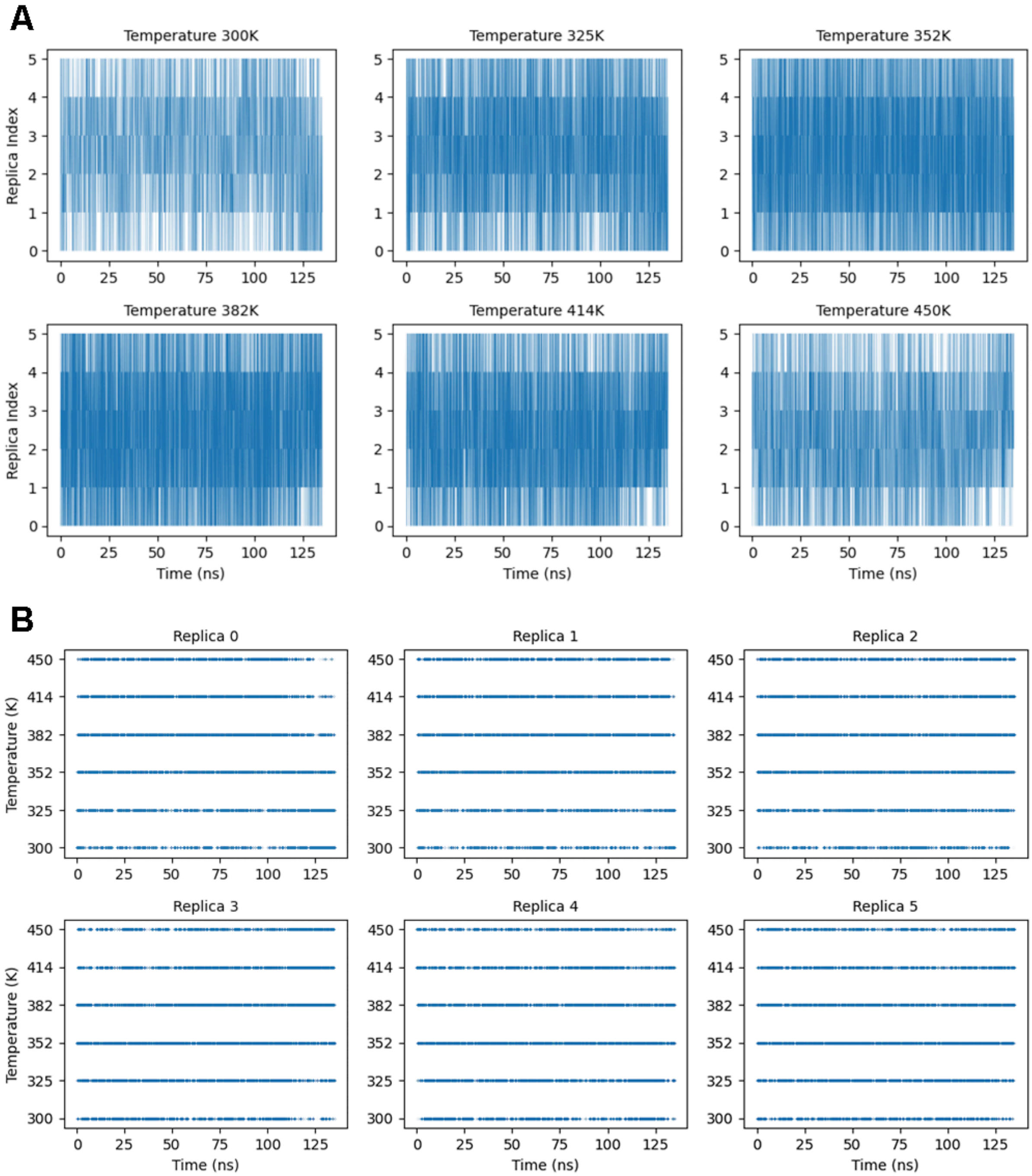

Figure S2. Replica exchange plots of the PT-WTE simulation. A) Replica index found at each selected temperature as a function of time. B) Temperature at which each individual replica is simulated as function of time. The average round trip time with its standard error is 0.573 ± 0.015 ns.

```
CLUSTAL O(1.2.4) multiple sequence alignment


sp|P26012|ITB8_HUMAN      KKYPVDLYYLVDVSASMHNNIEKLNSVGNDLSRKMAFFSRDFRLGFGSYVDKTVSPYISI
sp|P05556|ITB1_HUMAN      EDYPIDLYYLMDLSYSMKDDLENVKSLGTDLMNEMRRITSDFRIGFGSFVEKTVMPYIST
sp|P18564|ITB6_HUMAN      EDYPVDLYYLMDLSASMDDDLNTIKELGSRLSKEMSKLTSNFRLGFGSFVEKPVSPFVKT
sp|P05106|ITB3_HUMAN      EDYPVDIYYLMDLSYSMKDDLWSIQNLGTKLATQMRKLTSNLRIGFGAFVDKPVSPYMYI
sp|P18084|ITB5_HUMAN      EDYPVDLYYLMDLSLSMKDDLDNIRSLGTKLAEEMRKLTSNFRLGFGSFVDKDISPFSYT
                          :.**:*:***:*:* **.::: .:..:*. *  :*  :: ::*:***::*:* : *:

sp|P26012|ITB8_HUMAN      HPE-RIHNQCSDY--NLDCMPPHGYIHVLSLTENITEFEKAVHRQKISGNIDTPEGGFDA
sp|P05556|ITB1_HUMAN      TPA-KLRNPCTS---EQNCTSPFSYKNVLSLTNKGEVFNELVGKQRISGNLDSPEGGFDA
sp|P18564|ITB6_HUMAN      TPE-EIANPCSS--IPYFCLPTFGFKHILPLTNDAERFNEIVKNQKISANIDTPEGGFDA
sp|P05106|ITB3_HUMAN      SPPEALENPCY--DMKTTCLPMFGYKHVLTLTDQVTRFNEEVKKQSVSRNRDAPEGGFDA
sp|P18084|ITB5_HUMAN      APR-YQTNPCIGYKLFPNCVPSFGFRHLLPLTDRVDSFNEEVRKQRVSRNRDAPEGGFDA
                           *     * *        *   ..: ::* **:    *:: * .* :* * *:*******

sp|P26012|ITB8_HUMAN      MLQAAVCESHIGWRKEAKRLLLVMTDQTSHLALDSKLAGIVVPNDGNCHLK-NNVYVKST
sp|P05556|ITB1_HUMAN      IMQVAVCGSLIGWRNV-TRLLVFSTDAGFHFAGDGKLGGIVLPNDGQCHLE-NNMYTMSH
sp|P18564|ITB6_HUMAN      IMQAAVCKEKIGWRNDSLHLLVFVSDADSHFGMDSKLAGIVIPNDGLCHLDSKNEYSMST
sp|P05106|ITB3_HUMAN      IMQATVCDEKIGWRNDASHLLVFTTDAKTHIALDGRLAGIVQPNDGQCHVGSDNHYSAST
sp|P18084|ITB5_HUMAN      VLQAAVCKEKIGWRKDALHLLVFTTDDVPHIALDGKLGGLVQPHDGQCHLNEANEYTASN
                          ::*.:** . ****:   :**:. :*   *:. *.:*.*:* *:** **:   * *  *

sp|P26012|ITB8_HUMAN      TMEHPSLGQLSEKLIDNNINVIFAVQGKQFHWYKDLLPLLPGTIAGEIESKAANLNNLVV
sp|P05556|ITB1_HUMAN      YYDYPSIAHLVQKLSENNIQTIFAVTEEFQPVYKELKNLIPKSAVGTLSANSSNVIQLII
sp|P18564|ITB6_HUMAN      VLEYPTIGQLIDKLVQNNVLLIFAVTQEQVHLYENYAKLIPGATVGLLQKDSGNILQLII
sp|P05106|ITB3_HUMAN      TMDYPSLGLMTEKLSQKNINLIFAVTENVVNLYQNYSELIPGTTVGVLSMDSSNVLQLIV
sp|P18084|ITB5_HUMAN      QMDYPSLALLGEKLAENNINLIFAVTKNHYMLYKNFTALIPGTTVEILDGDSKNIIQLII
                            ::*::. : :** ::*:  ****  :    *::   *:* : .  :. .: *: :*::

sp|P26012|ITB8_HUMAN      EAYQKLIS
sp|P05556|ITB1_HUMAN      DAYNSLSS
sp|P18564|ITB6_HUMAN      SAYEELRS
sp|P05106|ITB3_HUMAN      DAYGKIR-
sp|P18084|ITB5_HUMAN      NAYNSIR-
                          .** .:
```

Figure S3. Multiple sequence alignment between the "head" residues (corresponding to β3 residues 107-352) of all the human RGD β-subunits (β1, β3, β5, β6, β8) performed with the ClustalOmega software.

```
CLUSTAL O(1.2.4) SDL sequence alignment

sp|P05556|ITB1_HUMAN      VMPYISTT-PAKLRNPCTSEQ---NCTSPFSY
sp|P18084|ITB5_HUMAN      ISPFSYTA-PRYQTNPCIGYKLFPNCVPSFGF
sp|P05106|ITB3_HUMAN      VSPYMYISPPEALENPCYDMKT--TCLPMFGY
sp|P26012|ITB8_HUMAN      VSPYISIH-PERIHNQCSDYNL--DCMPPHGY
sp|P18564|ITB6_HUMAN      VSPFVKTT-PEEIANPCSSIPYF--CLPTFGF
                          : *:     *    * * .      *   ..:
```

Figure S4. Multiple sequence alignment between the SDL residues (corresponding to β3 residues 161-192) of all the human RGD β-subunits (β1, β3, β5, β6, β8) performed with the ClustalOmega software.

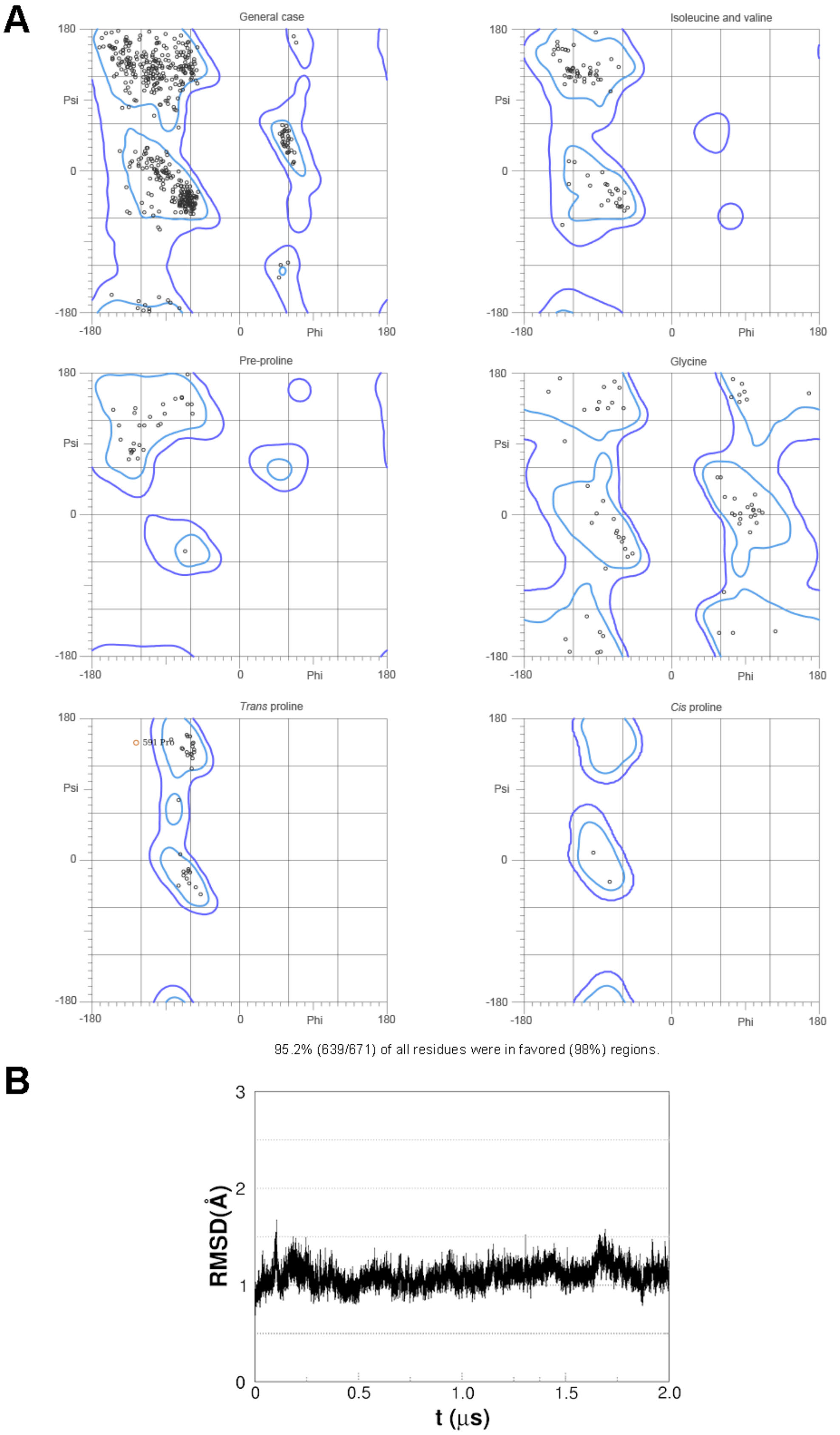

Figure S5. A) Ramachandran plot of the αvβ5 homology model and B) RMSD plot of the secondary structure element (Cα atoms) over the 2 μs long MD simulation of αvβ5 in complex with *i*RGD.

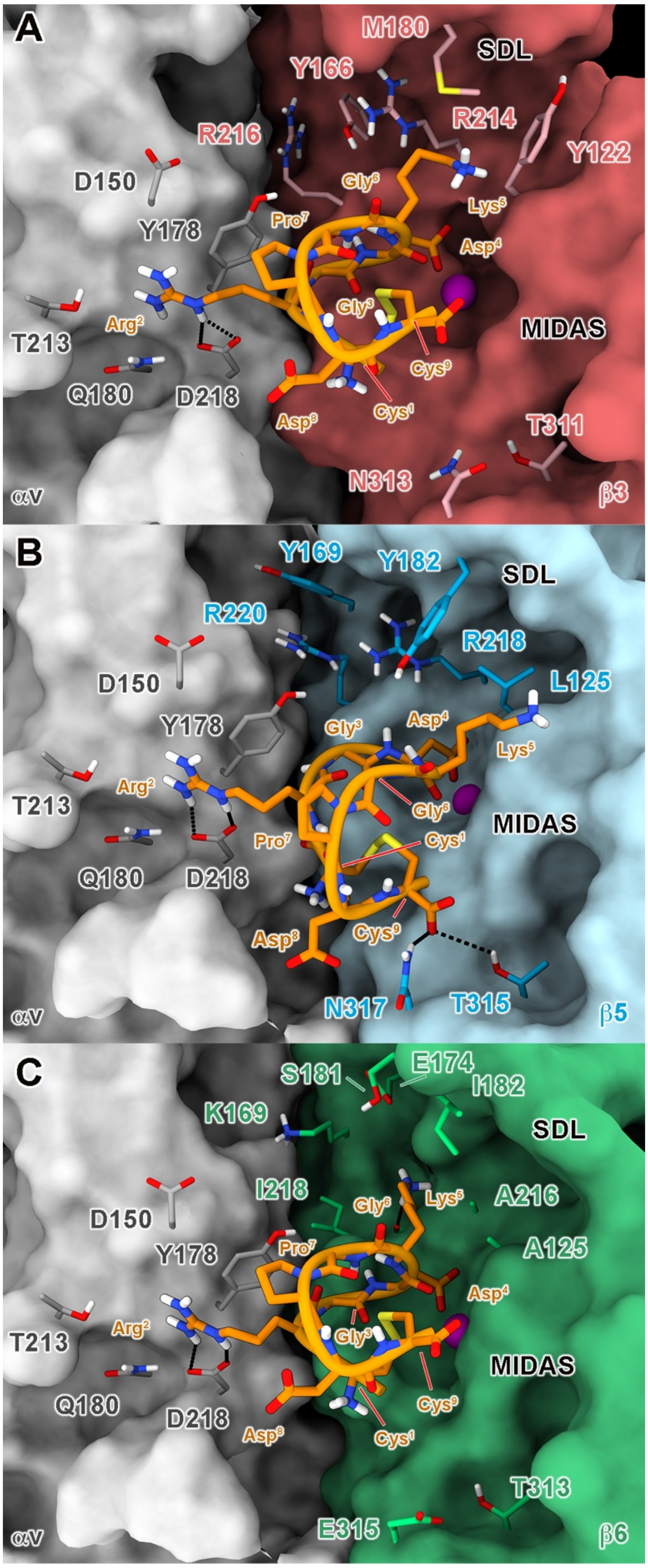

Figure S6. Docking-predicted binding mode of *i*RGD at the RGD binding site of αvβ3, αβ5 and αvβ6 integrins. The different receptors subunits are depicted as colored surfaces (αv=grey, β3=red, β5=cyan and β6=green). Amino acids important for peptide binding are highlighted as sticks, while the Mg^{2+} ion in the MIDAS is shown as a purple sphere. The ligand is represented as orange ribbon and sticks; nonpolar hydrogens ore omitted for sake of clarity; and H-bonds are shown as black dashed lines.

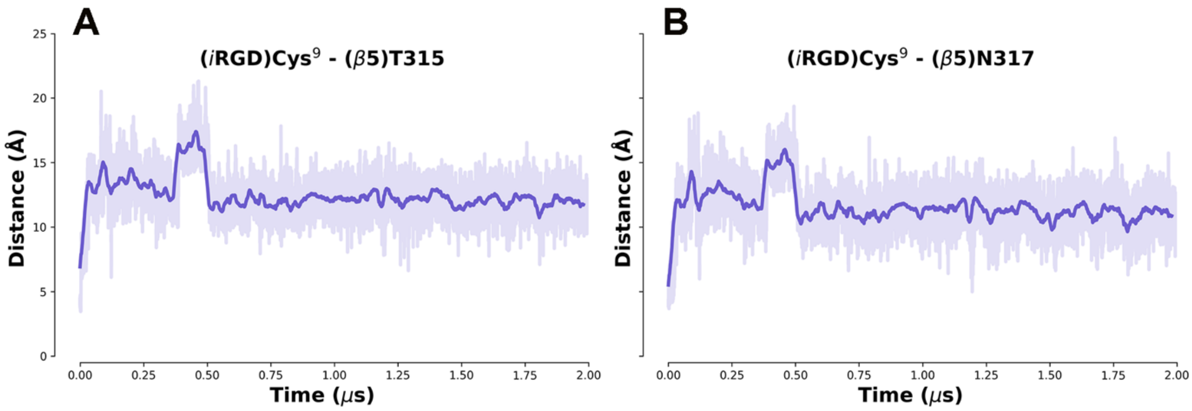

Figure S7. Interatomic distances between the C-ter carboxylic carbon of *i*RGD's Cys[9] with T315-Oγ[1] (A) and N317-Cγ (B). The bolded lines show values of the distance smoothed with a rolling window of 5 ns, while the actual fluctuations are shown with a slight transparency.

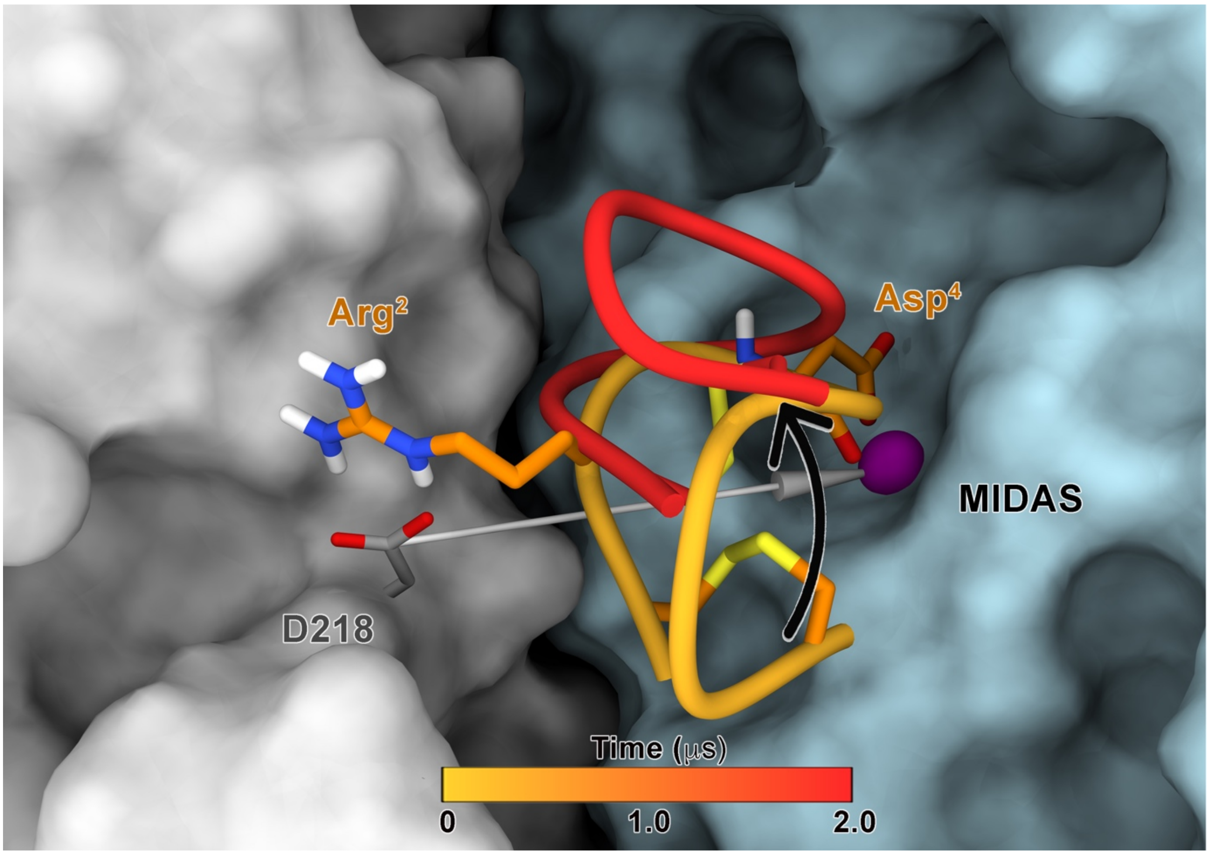

Figure S8. 3D representation of the upward rotation experienced by *i*RGD during the first stages of the MD simulation in complex with the αvβ5 receptor. The grey arrow represents the axis of the rotation. The receptor is depicted as light gray (αv subunit) and cyan (β5 subunit) surfaces. The ligand backbone is shown in orange (initial MD frame) and red (final MD frame) cartoons, while the sidechain of Arg[2] and Asp[4] are as shown as sticks to highlight the typical RGD binding pattern. The divalent Mg^{2+} cation at the MIDAS is depicted as a purple sphere.

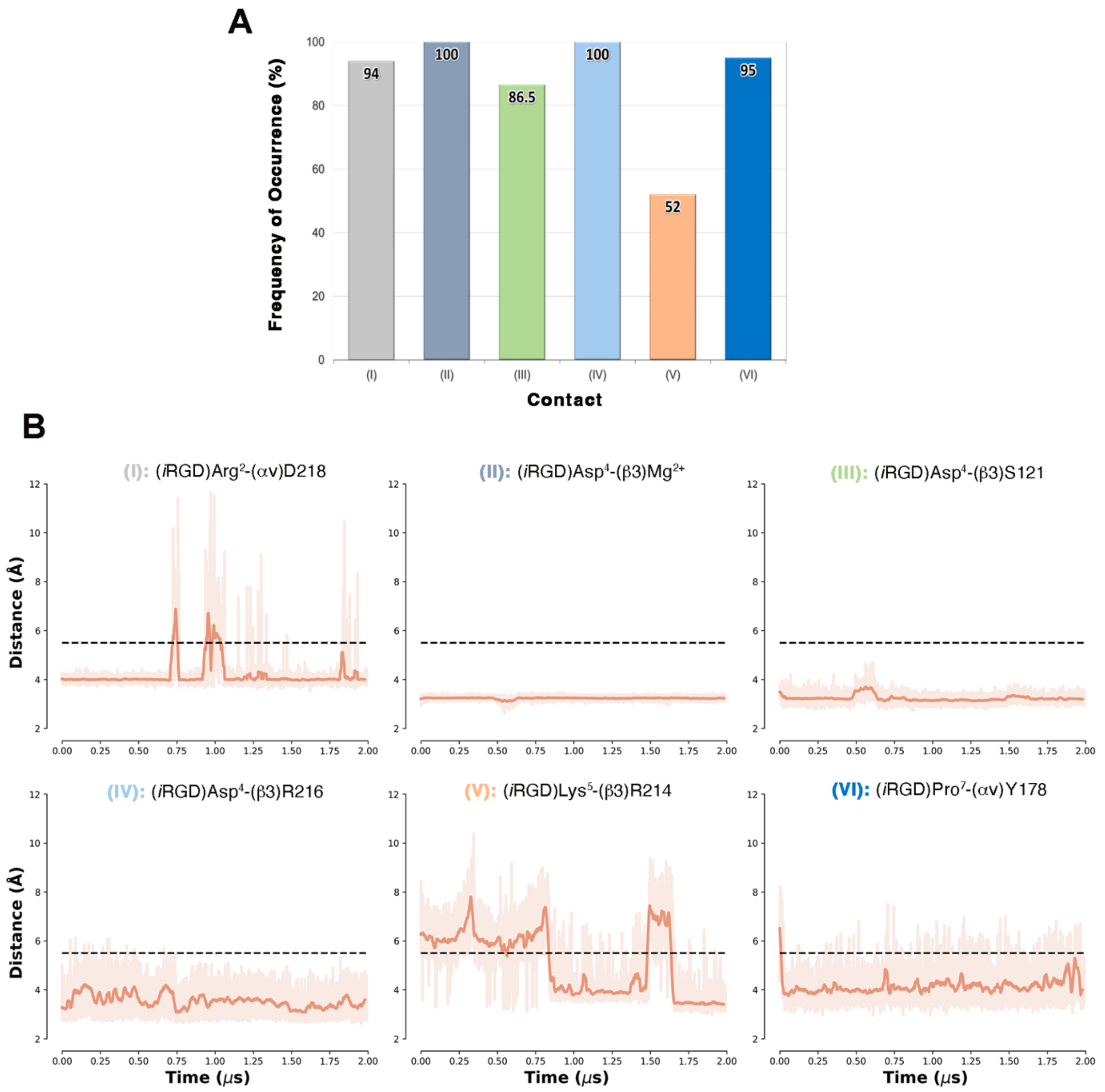

Figure S9. Analysis of the *i*RGD–αvβ3 residues interactions along the MD simulation. A) Frequency of Occurrence (% of collected frames in which the contact is formed) of the interatomic interactions: (I) Arg^2 (Cζ atom) – (αv)-D218 (Cγ atom); (II) Asp^4 (Cζ atom) – (β3)-Mg^{2+}; (III) Asp^4 (Cζ atom) – (β3)-S121 (Oγ atom); (IV) Asp^4 (backbone-N atom) – (β3)-R216 (backbone-O atom); (V) Lys^5 (backbone-O atom) – (β3)-R214 (Nζ atom); (VI) Pro^7 (Center of Mass of the pyrrolidine ring) – (αv)-Y178 (Center of Mass of the phenol ring) B) Evolution of the interatomic distances of contacts (I) – (VI) over the MD timescale. In each plot, the adopted cutoff (5.5 Å) is shown as a dashed black line.

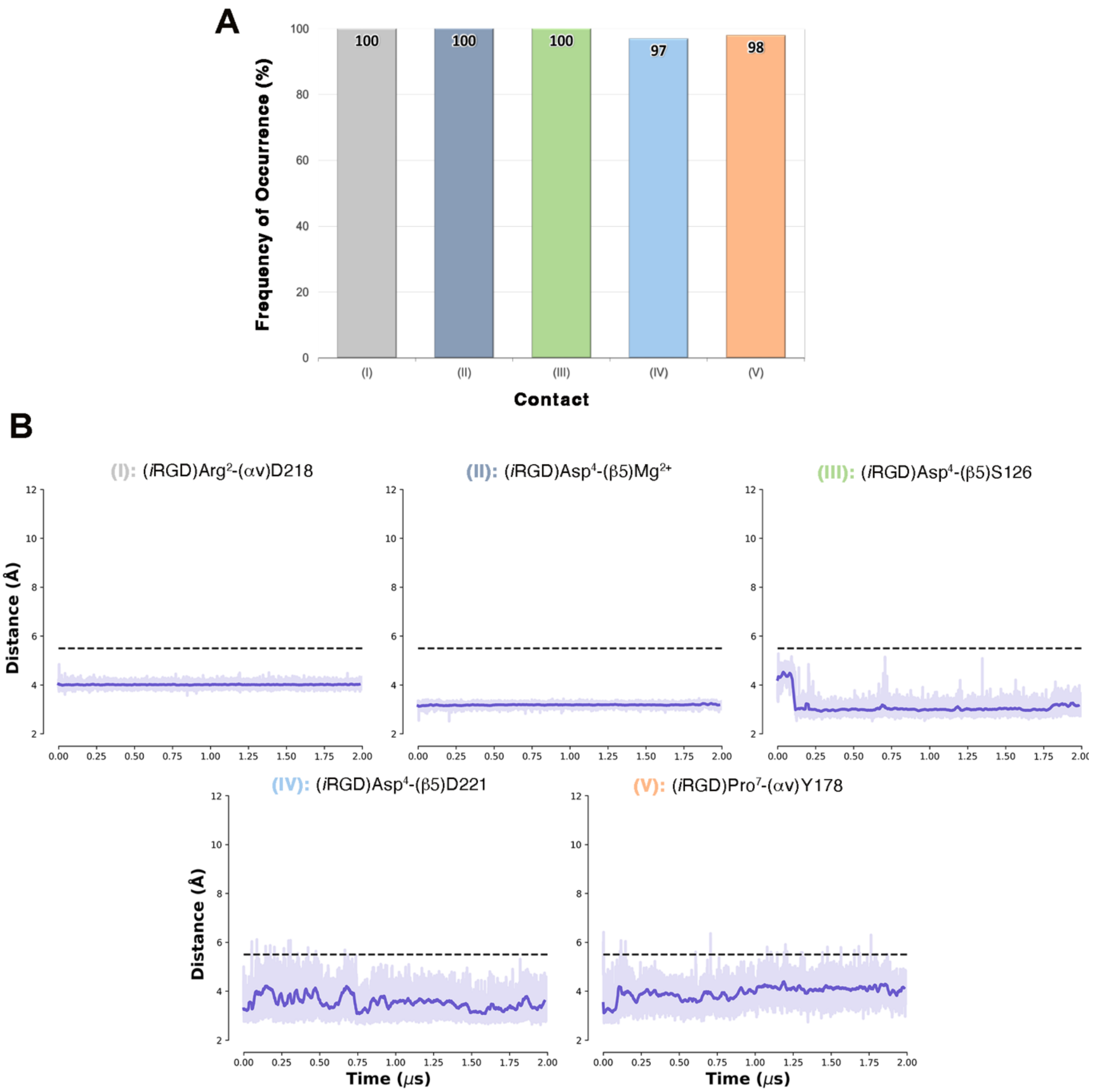

Figure S10. Analysis of the *i*RGD–αvβ5 residues interactions along the MD simulation. A) Frequency of Occurrence (% of collected frames in which the contact is formed) of the interatomic interactions: (I) Arg^2 (Cζ atom) – (αv)-D218 (Cγ atom); (II) Asp^4 (Cζ atom) – (β5)-Mg^{2+}; (III) Asp^4 (Cζ atom) – (β5)-S126 (backbone-N atom); (IV) Asp^4 (backbone-N atom) – (β5)-D221 (backbone-O atom); (V) Pro^7 (Center of Mass of the pyrrolidine ring) – (αv)-Y178 (Center of Mass of the phenol ring) B) Evolution of the interatomic distances of contacts (I) – (V) over the MD timescale. In each plot, the adopted cutoff (5.5 Å) is shown as a dashed black line.

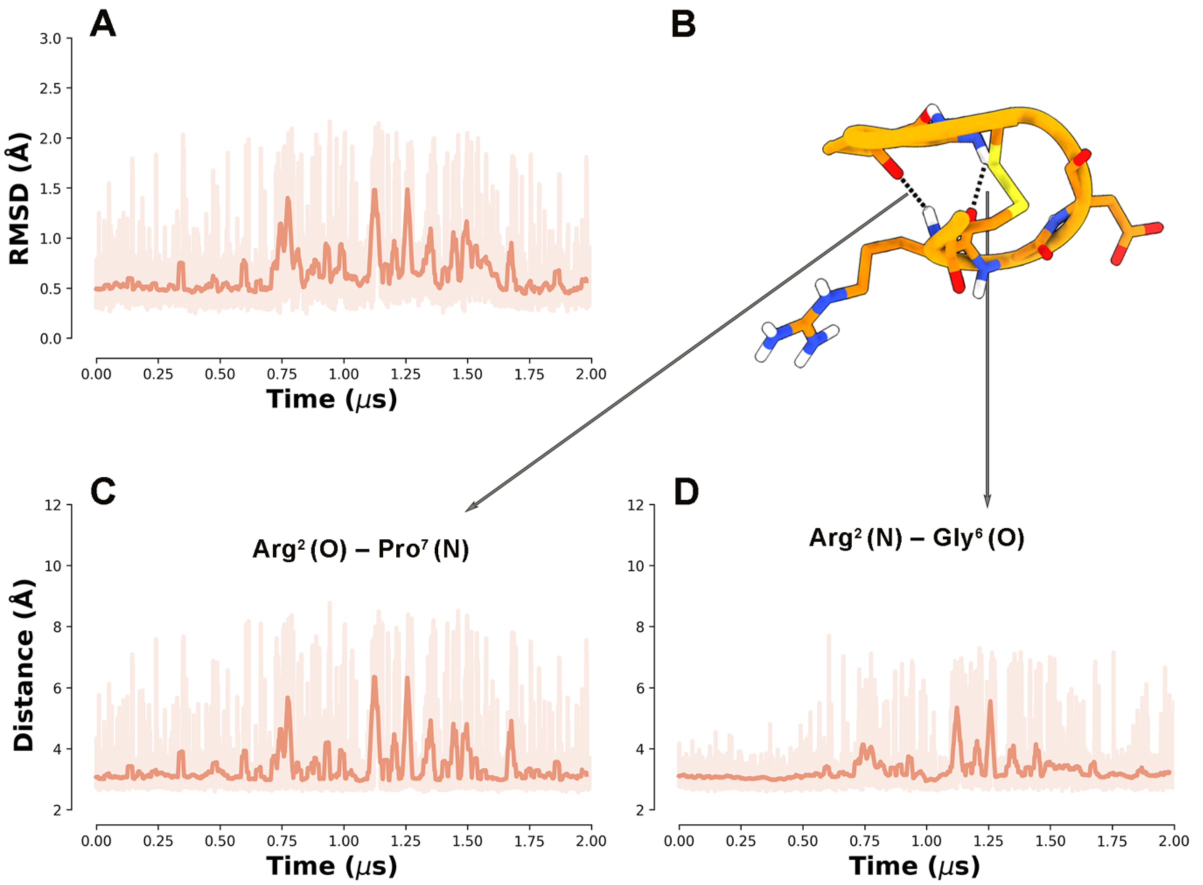

Figure S11. A) RMSD plot of the backbone atoms of *i*RGD in complex with αvβ3 computed respect to the PT-WTE-predicted conformation of the peptide (B). Stability of the two intramolecular H-bonds (C and D) found in PT-WTE between Arg^{2} (C-O)-Gly^{6} (N-H) and Arg^{2} (N-H)-Pro^{7} (C-O), respectively.

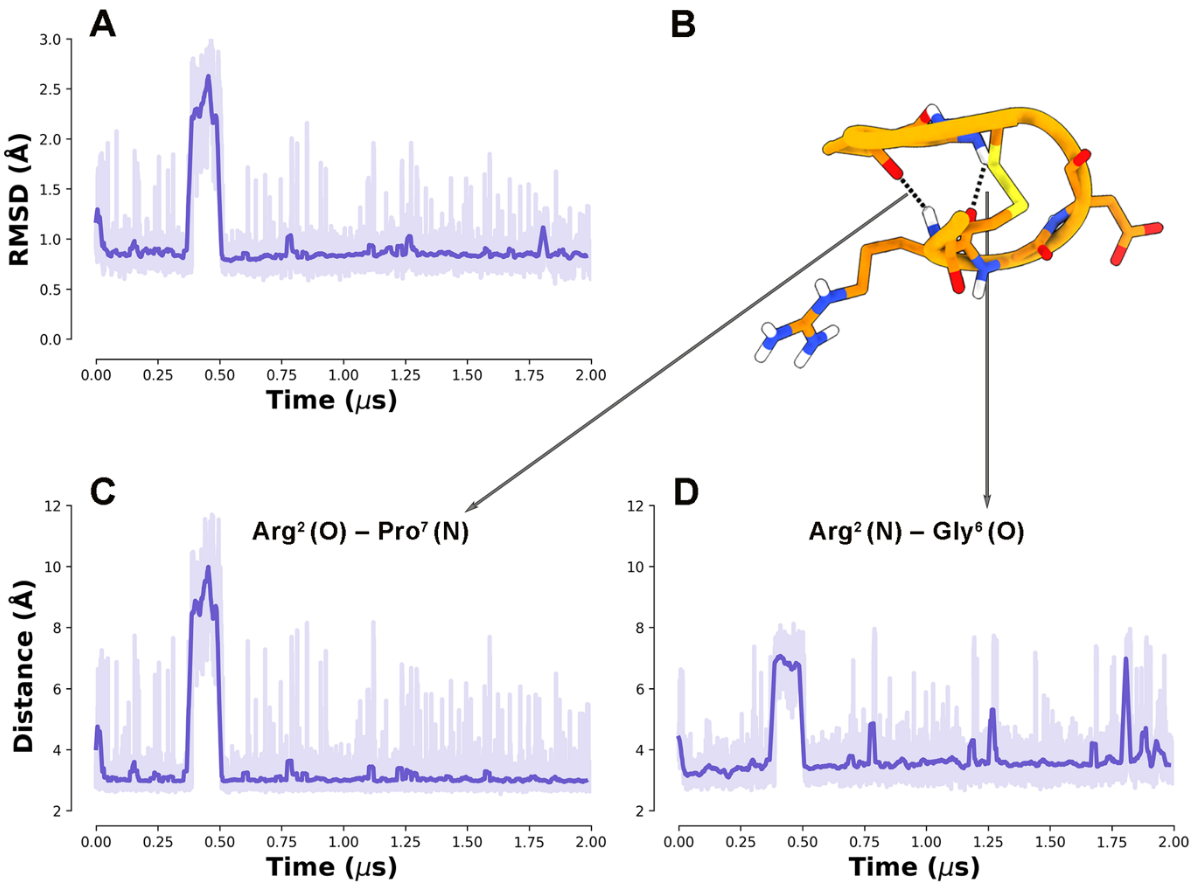

Figure S12. A) RMSD plot of the backbone atoms of *i*RGD in complex αvβ5 computed respect to the PT-WTE-predicted conformation of the peptide (B). Stability of the two intramolecular H-bonds (C and D) found in PT-WTE between Arg^2 (C-O)-Gly^6 (N-H) and Arg^2 (N-H)-Pro^7 (C-O), respectively.

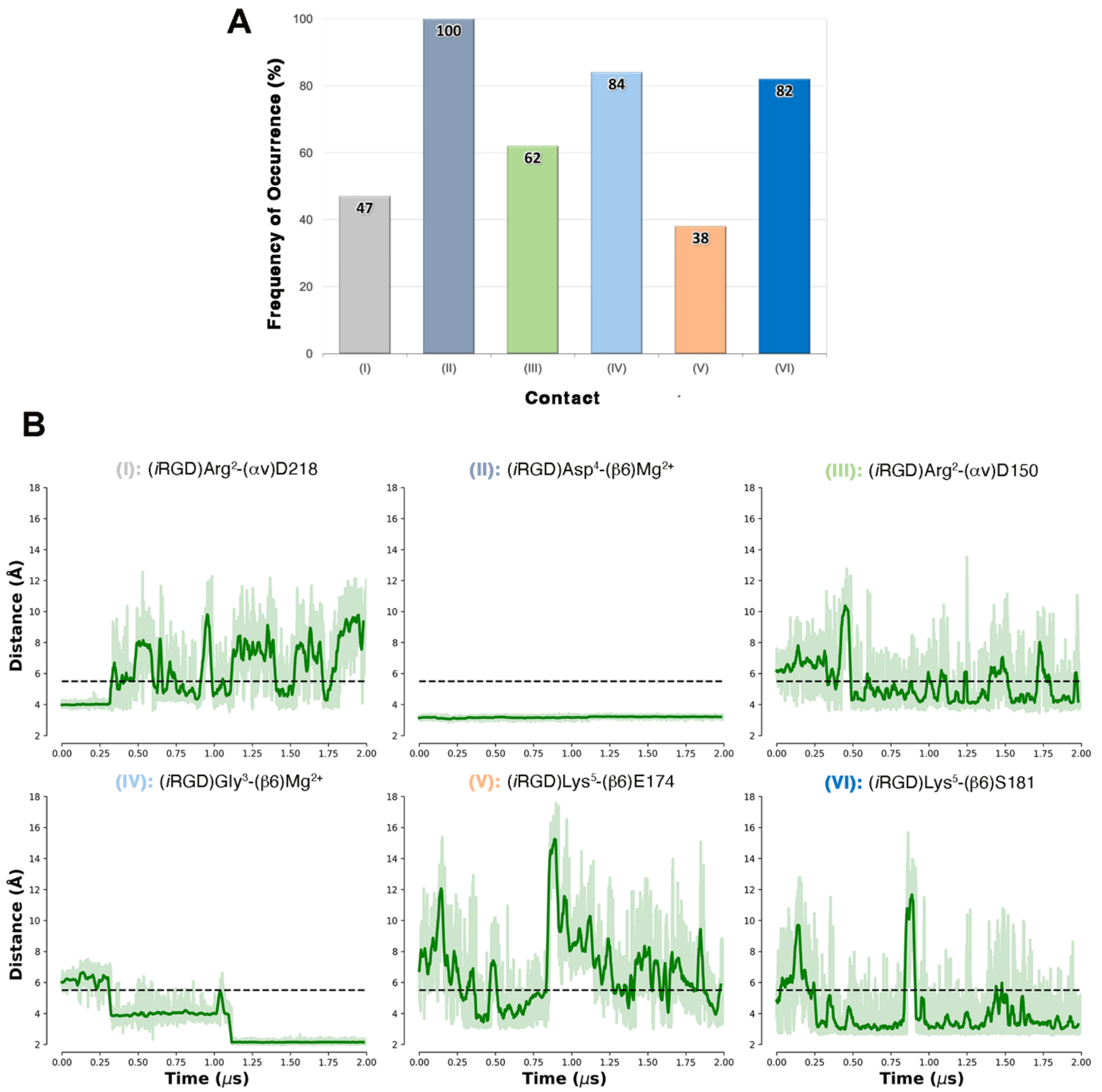

Figure S13. Analysis of the *i*RGD–αvβ6 residues interactions along the MD simulation. A) Frequency of Occurrence (% of collected frames in which the contact is formed) of the interatomic interactions: (I) Arg^2 (Cζ atom) – (αv)-D218 (Cγ atom); (II) Asp^4 (Cζ atom) – (β6)-Mg^{2+}; Arg^2 (Cζ atom) – (αv)-D150 (Cγ atom); (IV) Gly^3 (backbone-O atom) – (β6)-Mg^{2+}; (V) Lys^5 (Nζ) – (β6)-E174 (Cε-atom); (VI) Lys^5 (Nζ) – (β6)-S181 (Oγ atom) B) Evolution of the interatomic distances of contacts (I) – (VI) over the MD timescale. In each plot, the adopted cutoff (5.5 Å) is shown as a dashed black line.

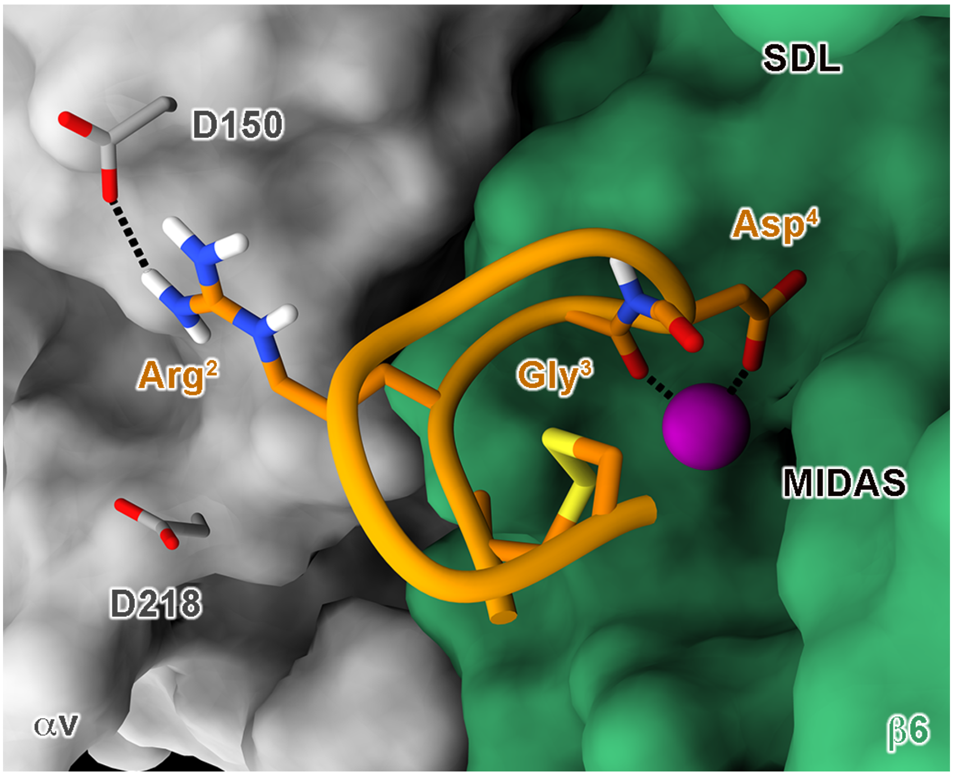

Figure S14. 3D representation of the unusual Mg^{2+}-chelation scheme and binding pattern experienced by *i*RGD in the αvβ6 receptor. The receptor is depicted as light gray (αv subunit) and green (β6 subunit) surfaces. The ligand backbone is shown in orange (initial MD frame) cartoons, while the sidechain of Arg^2 and Asp^4 are as shown as sticks to highlight the loss of typical RGD binding pattern: the interaction of Arg^2 with (αv)-D218 is lost and replaced by a salt-bridge with (αv)-D150, while the Mg^{2+} cation (purple sphere) is chelated by both the Asp^4 carboxylate and the backbone carbonyl of Gly^2, leading to a distortion in the backbone conformation of the peptide.

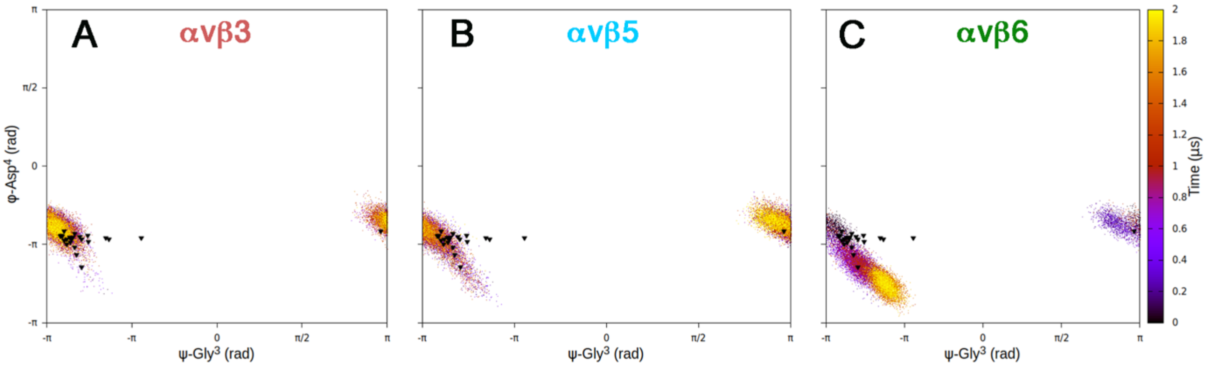

Figure S15. Comparison of the dihedral values assumed by *i*RGD's ϕ-Gly*3* and ψ-Asp^4 in the three MD trajectories (A, B, C) with all the available experimental structures of RGD peptides in complex with RGD-integrin receptors. In each plot, the torsion values observed during the simulations are shown as dots colored based on their timestep. The ϕ-Gly^3 and ψ-Asp^4 values measured in the experimental structures are depicted as black triangle markers. The list of the PDBs used for the analysis is the following: 2VDM, 2VDN, 2VDO, 2VDP 2VDQ, 2VDR, 3ZDY, 3ZDZ, 3ZE0, 3ZE1, 3ZE2, 4WK4, 4WK2, 4WK0, 3VI4, 4MMZ, 4MMY, 4MMX, 1L5G, 6MK0, 6MSL, 4UM9, 5FFO.

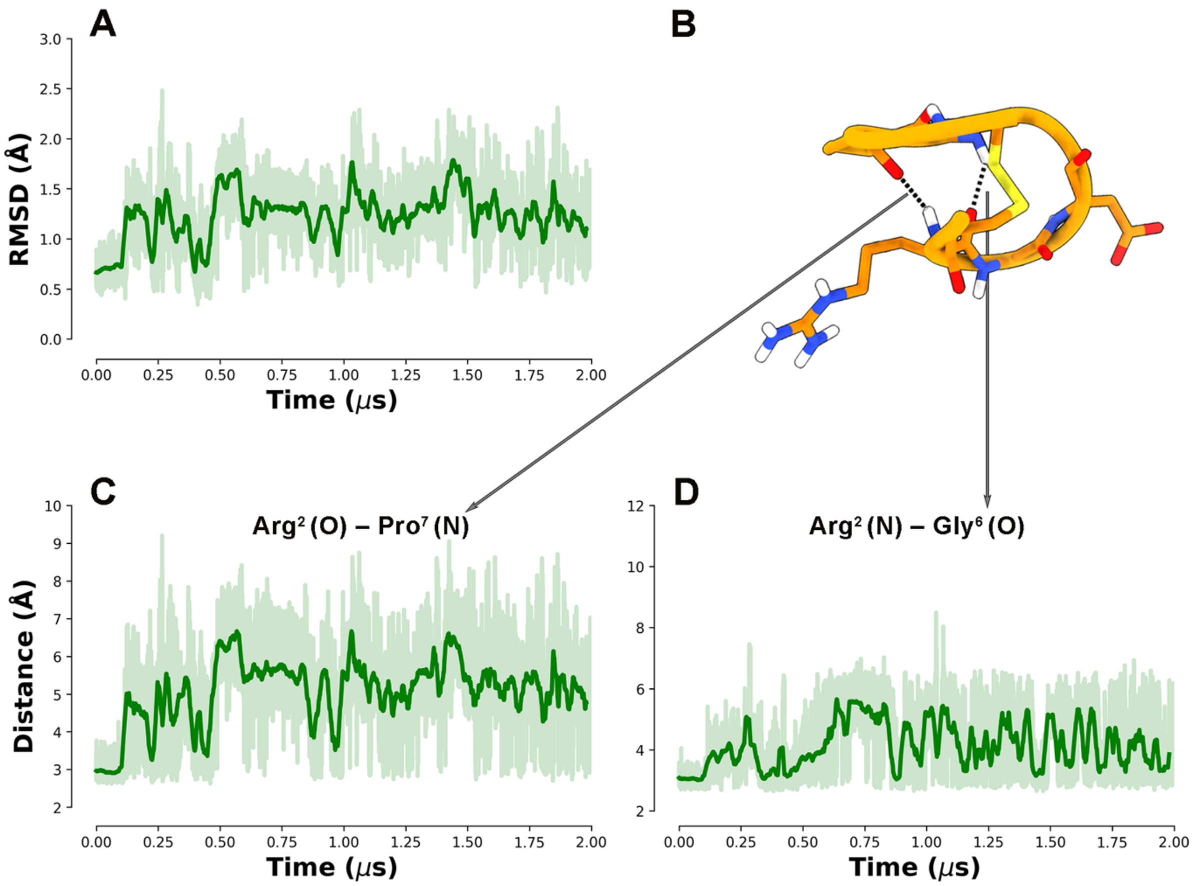

Figure S16. A) RMSD plot of the backbone atoms of *i*RGD in complex with αvβ6 computed respect to the PT-WTE-predicted conformation of the peptide (B). Stability of the two intramolecular H-bonds (C and D) found in PT-WTE between Arg^2 (C-O)-Gly^6 (N-H) and Arg^2 (N-H)-Pro^7 (C-O), respectively.

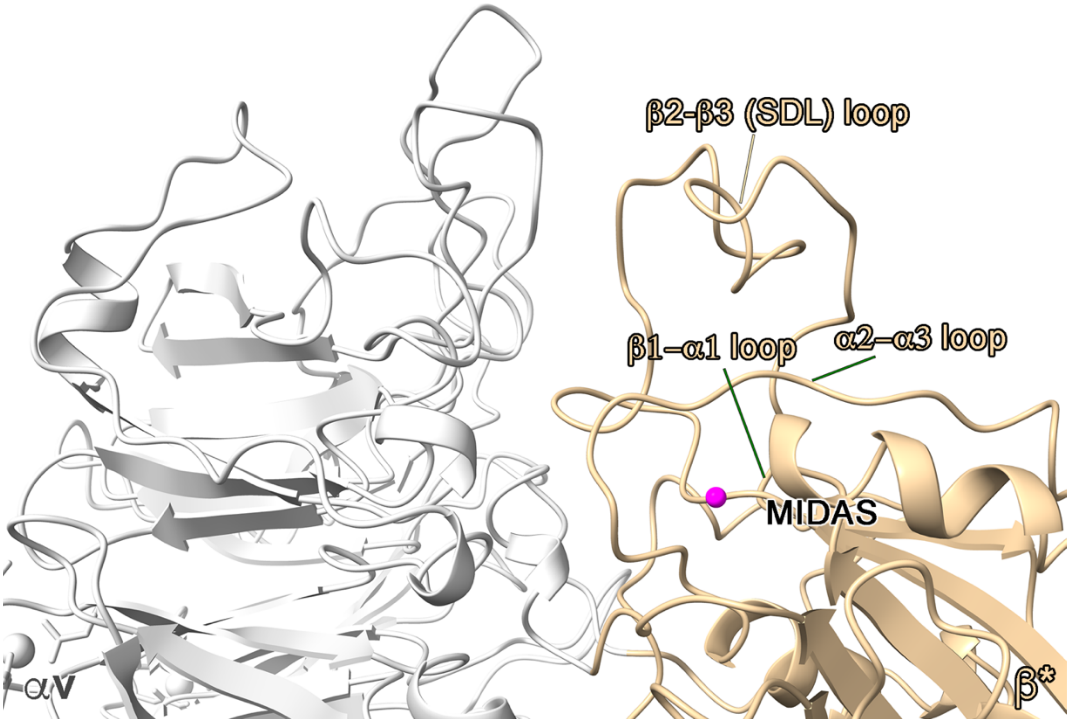

Figure S17. Schematic representation of the secondary structure elements of the integrins RGD binding site and SDL cavity. αv subunit is shown as gray cartoon while a generic β* subunit is shown in beige.

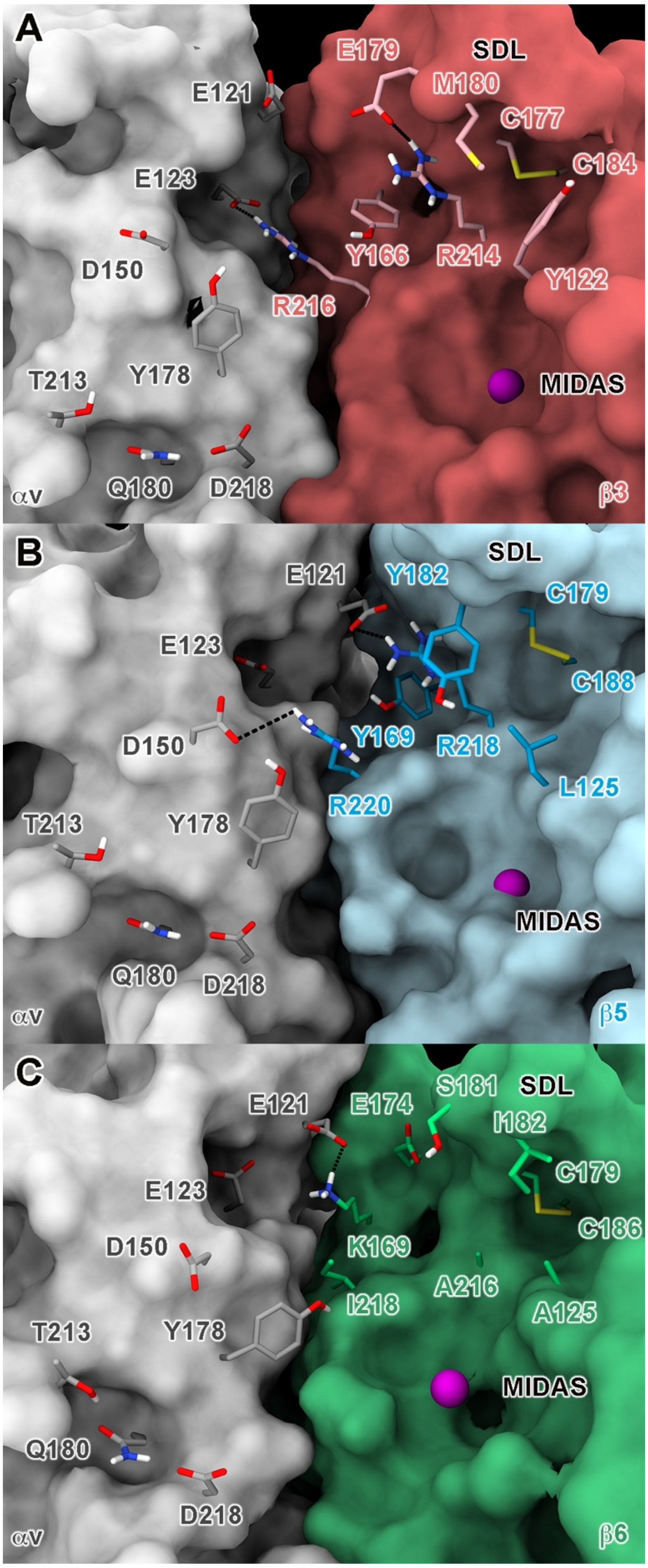

Figure S18. 3D representation of the RGD binding site of αvβ3 (A), αvβ5 (B) and αvβ6 (C) receptors. The most important mutations occurring at the SDL subpocket were highlighted in sticks. The different receptors subunits are depicted as colored surfaces (αv=grey, β3=red, β5=cyan and β6=green).

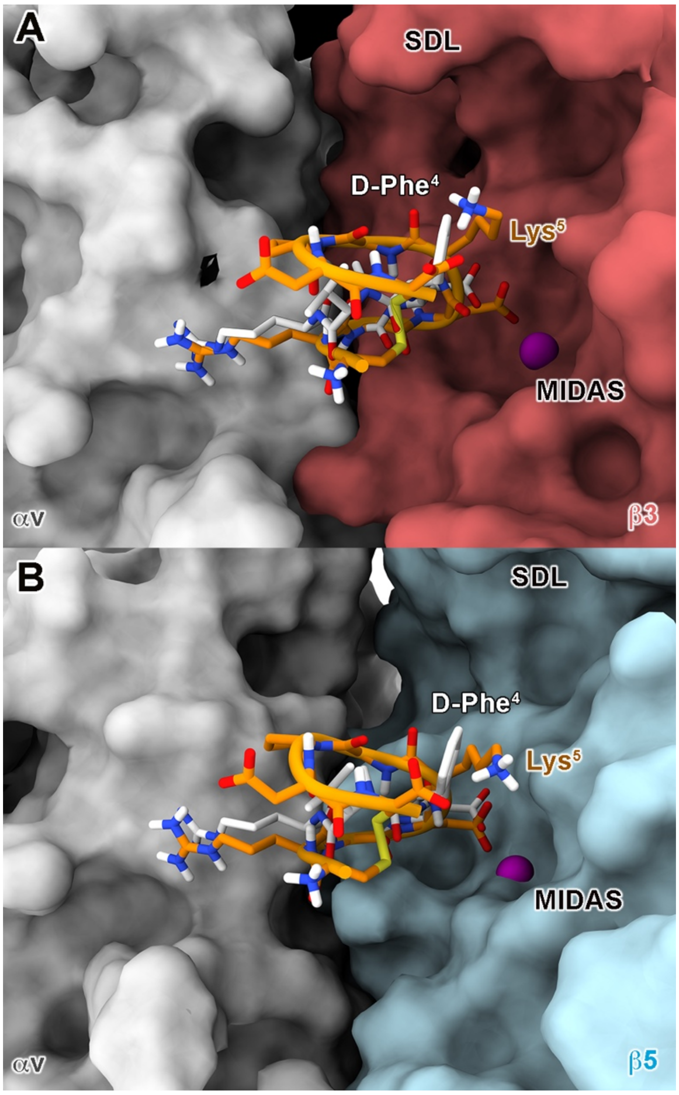

Figure S19. Superposition of the crystal structure of cilengitide at αvβ3 (PDB code: 1L5G) with the MD-predicted binding pose of *i*RGD at αvβ3 (A) and αvβ5 (B). *i*RGD is shown as orange sticks and ribbon, while cilengitide is colored in white. The different receptors subunits are depicted as colored surfaces (αv=grey, β3=red, β5=cyan).

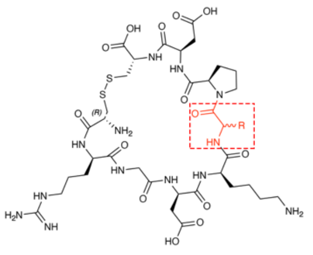

Compound	X
1 (iRGD)	L-Glycine
3	L-Valine
4	L-Leucine
5	L-Isoleucine
6	L-Phenylalanine
7	L-Tryptophan
8	L-Cyclohexylglycine
9	L-Cyclohexylalanine
10	L-Allylglycine
11	L-Cyclopropylalanine

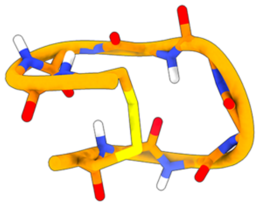

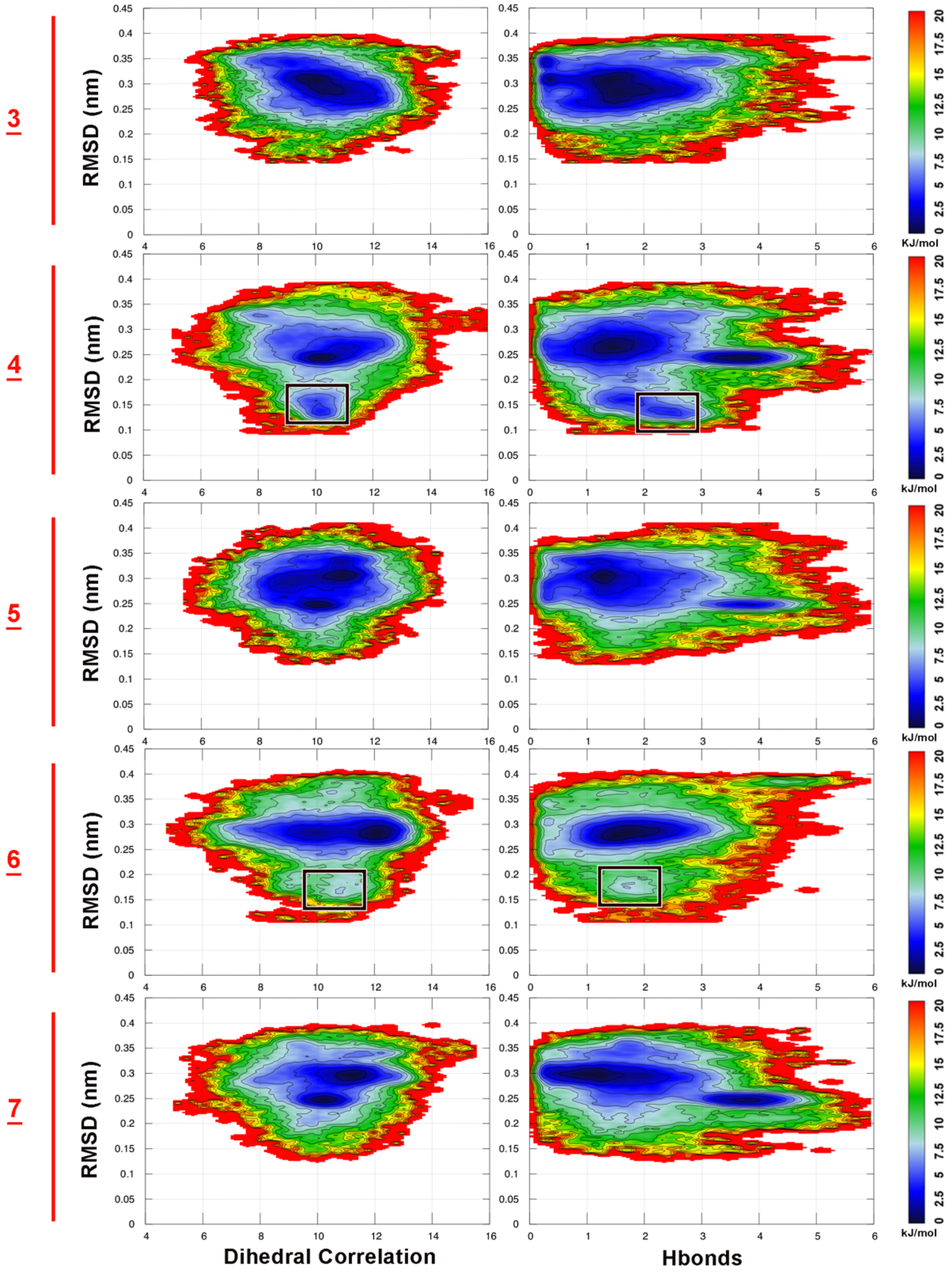

Figure S20. Results of the PT-WTE calculations on the designed compounds **3-7**. All the shown FES were computed after 150 ns (per replica) of simulation. As for the parent peptide **1,** in all the cases metadynamics converged after about 80-100 ns. Convergence was estimated as described in the Materials and Methods section for compound **1**. The average exchange acceptance ratio was ≈ 25%.

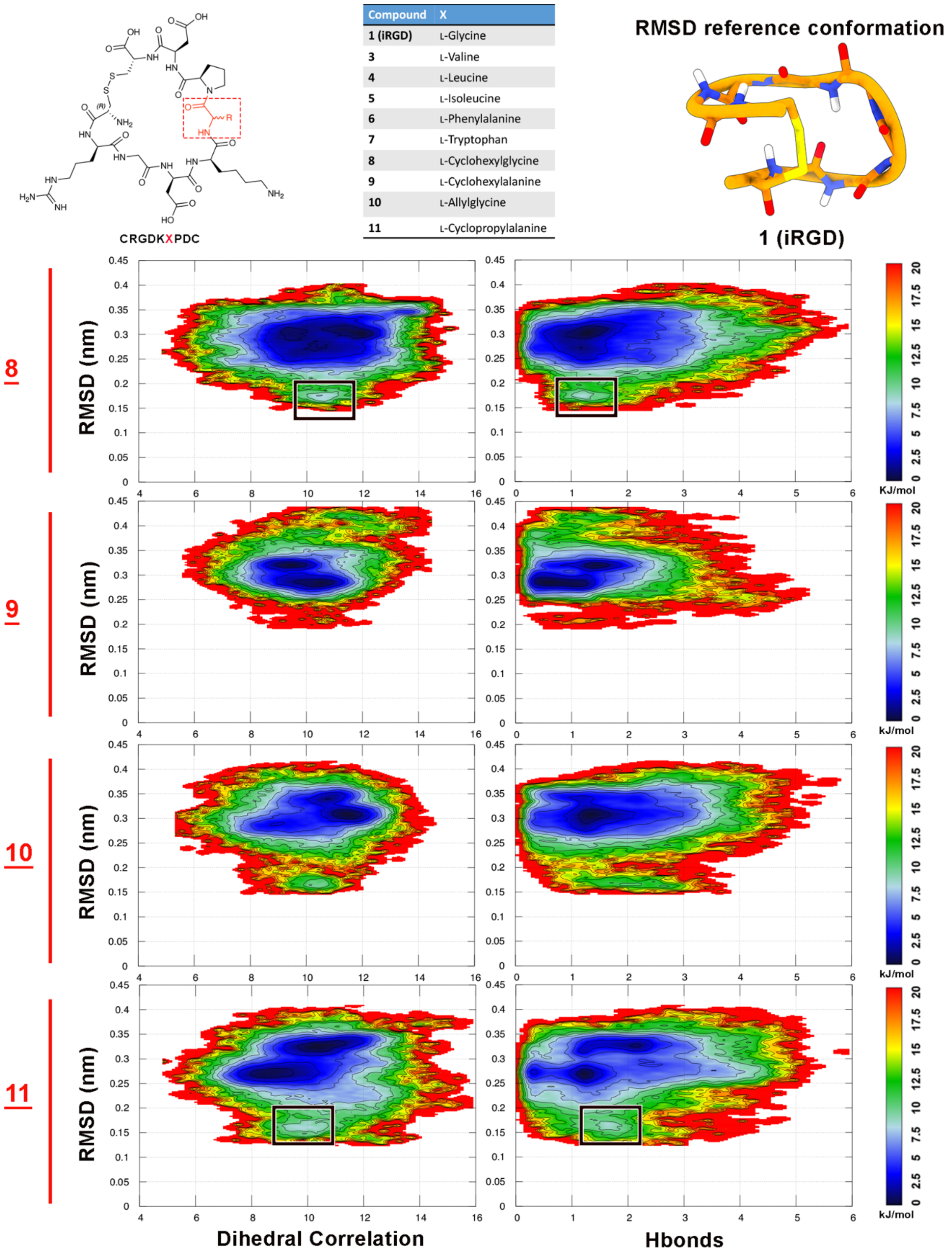

Compound	X
**1 (iRGD)**	L-Glycine
**3**	L-Valine
**4**	L-Leucine
**5**	L-Isoleucine
**6**	L-Phenylalanine
**7**	L-Tryptophan
**8**	L-Cyclohexylglycine
**9**	L-Cyclohexylalanine
**10**	L-Allylglycine
**11**	L-Cyclopropylalanine

Figure S21. Results of the PT-WTE calculations on the designed compounds **8-11.** All the shown FES were computed after 150 ns (per replica) of simulation. As for the parent peptide **1**, in all the cases metadynamics converged after about 80-100 ns. Convergence was estimated as described in the Materials and Methods section for compound **1**. The average exchange acceptance ratio was ≈ 25%.
